# Metabolic syndrome as an independent predictor of response and survival in locally advanced gastric cancer treated with neoadjuvant immunochemotherapy

**DOI:** 10.3389/fonc.2026.1842129

**Published:** 2026-07-21

**Authors:** Fang Li, Honghai Guo, Jiaxiang Wu, Haotian Wu, Zhiqiang Wang, Xiaolong Li, Zhenjiang Guo, Yuan Tian, Yihao Yang, Shuo Ma, Wenqian Ma, Qun Zhao

**Affiliations:** 1 Department of Pathology, The Fourth Hospital of Hebei Medical University, Shijiazhuang, Hebei, China; 2 The Third Department of Surgery, the Fourth Hospital of Hebei Medical University, Shijiazhuang, Hebei, China; 3 Hebei Key Laboratory of Precision Diagnosis and Comprehensive Treatment of Gastric Cancer, The Fourth Hospital of Hebei Medical University, Shijiazhuang, Hebei, China; 4 Big Data Analysis and Mining Application for Precise Diagnosis and Treatment of Gastric Cancer Hebei Provincial Engineering Research Center, The Fourth Hospital of Hebei Medical University, Shijiazhuang, Hebei, China; 5 Department of General Surgery, Shijiazhuang People’s Hospital, Shijiazhuang, Hebei, China; 6 Department of General Surgery, Baoding Central Hospital, Baoding, Hebei, China; 7 Department of General Surgery, Hengshui People’s Hospital, Hengshui, Hebei, China; 8 Department of Endoscopy, The Fourth Hospital of Hebei Medical University, Shijiazhuang, China

**Keywords:** immune-related adverse events, locally advanced gastric cancer, metabolic syndrome, neoadjuvant immunotherapy, obesity paradox, pathological complete response

## Abstract

**Background:**

Metabolic syndrome (MetS) is prevalent in gastric cancer patients. The obesity paradox, where metabolic excess is associated with improved outcomes in patients receiving immune checkpoint inhibitor (ICI) therapy, remains uninvestigated in locally advanced gastric cancer (LAGC) treated with neoadjuvant immunochemotherapy. We evaluated MetS as a dual predictor of pathological response, immune-related adverse events (irAEs), disease-free survival (DFS), and overall survival (OS).

**Methods:**

This multicenter retrospective study included 680 LAGC patients treated with neoadjuvant PD-1 inhibitor plus chemotherapy followed by D2 gastrectomy. MetS was defined using NCEP-ATP III criteria, with East Asian-specific waist-circumference cut-points (≥90 cm men, ≥80 cm women) evaluated as a prespecified sensitivity analysis. Logistic regression, restricted cubic spline (RCS), Kaplan–Meier analysis, multivariable Cox regression for DFS and OS, and decision curve analysis were performed, and a MetS-inclusive nomogram was developed for pathological complete response (pCR) prediction.

**Results:**

MetS was present in 157/680 patients (23.1%). After multivariable adjustment for age, sex, BMI, cT stage, cN stage, PD-L1 CPS, MSI status, TyG group, and SII–PNI score, MetS was independently associated with higher pCR (adjusted OR ,  1.92, 95% CI 1.06–3.48, P ,  0.031) and major pathological response (MPR; OR ,  1.58, 95% CI 1.02–2.44, P ,  0.040). These associations were materially unchanged after additional adjustment for chemotherapy regimen, PD-1 agent, treatment cycle number, and study center. A positive dose-response trend was observed across MetS component strata (P-trend for pCR, 0.005; for MPR ,  0.001). Kaplan-Meier analysis showed superior DFS (log-rank P ,  0.017) and OS (log-rank P ,  0.019) in MetS patients. Multivariable Cox regression confirmed MetS as an independent favorable DFS predictor (HR ,  0.72, 95% CI 0.54–0.96, P ,  0.025) and a parallel multivariable Cox model for OS confirmed an independent favorable OS effect (adjusted HR ,  0.73, 95% CI 0.55-0.97, P ,  0.032). The MetS-inclusive nomogram achieved a bootstrap-corrected AUC of 0.76 for pCR prediction versus 0.64 for the clinical staging model (P ,  0.006); bootstrap calibration showed acceptable agreement (Hosmer–Lemeshow P ,  0.35), and decision curve analysis demonstrated greater net benefit than the clinical baseline across threshold probabilities of 5–25%.

**Conclusions:**

MetS independently predicts improved pathological response and survival without increasing immunotherapy toxicity in LAGC, extending the obesity paradox to the MetS phenotype and supporting an immunometabolic priming effect on ICI efficacy.

## Introduction

Gastric cancer remains the fourth most deadly cancer globally, with the highest incidence and mortality concentrated in East Asian populations, particularly China (1). Locally advanced gastric cancer (LAGC), defined as clinical stage cT2–4bNanyM0, represents over 60% of Chinese gastric cancer diagnoses and constitutes the primary candidate population for neoadjuvant systemic therapy followed by radical resection (2). The perioperative paradigm has evolved progressively from epirubicin-based triplet chemotherapy (ECF/MAGIC) to the docetaxel-containing FLOT regimen, with each successive refinement improving pathological complete response (pCR) rates and long-term outcomes (3).

The incorporation of PD-1/PD-L1 immune checkpoint inhibitors (ICIs) into perioperative chemotherapy backbones represents the most transformative recent advance in LAGC management. Registration trials including DANTE, MATTERHORN, KEYNOTE-585, and multiple single-arm Phase II studies have collectively established that PD-1 inhibitor addition raises pCR rates to 12–24%, increases R0 resection rates, and is associated with evolving disease-free and overall survival benefits (4–6). Despite this encouraging aggregate efficacy, individual response to immunotherapy remains strikingly heterogeneous, and the molecular underpinnings of this variability, particularly those related to host constitutional factors, remain incompletely characterized.

Metabolic syndrome (MetS), a cluster of interconnected metabolic abnormalities including central obesity, hypertriglyceridemia, reduced high-density lipoprotein cholesterol (HDL-C), elevated blood pressure, and impaired fasting glucose, affects approximately 25% of the global adult population and 20–30% of gastric cancer patients in Chinese series (7). MetS exerts diverse and often contradictory influences on cancer biology: it promotes tumorigenesis through chronic systemic inflammation, hyperinsulinemia, and pro-angiogenic adipokine secretion, yet may paradoxically enhance anti-tumor immunity when ICIs are employed (8).

The obesity paradox refers to the counterintuitive observation that overweight or obese cancer patients receiving ICIs achieve superior survival compared with their lean counterparts, and this phenomenon was first robustly documented in advanced melanoma by McQuade et al. (9) and has since been replicated across non-small cell lung cancer, renal cell carcinoma, and urothelial carcinoma. Proposed mechanisms include leptin-mediated upregulation of PD-1/PD-L1 expression providing an amplified checkpoint target for anti-PD-1 antibodies, visceral adipose tissue-driven pro-inflammatory cytokine milieu augmenting tumor immunogenicity, and adipose tissue-resident immune cell pools contributing to systemic T-cell activation (10, 11). However, all prior obesity paradox studies have focused on advanced-stage, palliative ICI settings, and it remains unknown whether a fully constituted MetS phenotype, as opposed to BMI alone, can similarly predict response in the neoadjuvant setting, where pathological response is directly measurable and treatment is administered with curative intent.

Additionally, a critical clinical counterquestion is whether MetS predisposes patients to higher rates or severity of irAEs. Existing evidence on this question is contradictory, with some studies reporting that metabolic comorbidities amplify irAE risk through systemic immunoactivation, while others suggest no significant association (12, 13). Resolving this dual predictor question, specifically whether metabolic syndrome simultaneously enhances treatment efficacy and toxicity or selectively enhances one without affecting the other, is of substantial clinical relevance for treatment decision-making in patients with locally advanced gastric cancer and coexisting metabolic disease.

This multicenter retrospective cohort study of 680 LAGC patients aimed to: (1) characterize MetS prevalence and component distribution in this population; (2) evaluate MetS status and MetS component score as predictors of pCR and MPR; (3) identify metabolic ‘sweet spots’ through RCS analysis of individual MetS components; (4) compare irAE incidence and severity between MetS and Non-MetS groups; (5) assess MetS as an independent prognostic factor for DFS and OS, with parallel multivariable Cox regression for both endpoints; and (6) construct a MetS-inclusive nomogram for individualized pCR prediction, with formal calibration assessment, decision curve analysis, and DeLong-test comparison against a clinical staging baseline.

## Materials and methods

### Study design and patient population

This multicenter retrospective cohort study enrolled patients from five tertiary hospitals in Hebei Province, China: The Fourth Hospital of Hebei Medical University (center 1), The First Hospital of Shijiazhuang (center 2), Shijiazhuang People’s Hospital (center 3), Baoding Central Hospital (center 4), and Hengshui People’s Hospital (center 5). Consecutive patients with histologically confirmed gastric or gastroesophageal junction adenocarcinoma who were treated between January 2019 and December 2024 were screened. The study was approved by the Institutional Review Board of each participating center and conducted in accordance with the Declaration of Helsinki. Patient enrolment, neoadjuvant immunochemotherapy administration, surgical management, and pathology workflow were governed by a single multi-center protocol developed by the Hebei Cancer Hospital Consortium and harmonised across all five participating tertiary cancer centers. All five centers employed the same three neoadjuvant chemotherapy backbones (SOX, XELOX, or FLOT) combined with one of three Chinese-approved PD-1 inhibitors (Sintilimab, Tislelizumab, or Camrelizumab); the cross-center distribution of chemotherapy backbones was comparable (SOX 43.1-54.9%, XELOX 26.0-34.7%, FLOT 14.8-25.7%), as was the cross-center distribution of PD-1 agents (Sintilimab 34.4-50.0%, Tislelizumab 30.3-38.1%, Camrelizumab 18.5-28.2%). The median number of preoperative PD-1 cycles was four at every center (center-level means 3.71-3.85), and all surgical procedures were planned D2 gastrectomies performed by board-certified gastric-cancer surgeons. Standardised reporting templates for surgical pathology, TRG grading, ypT/ypN staging, MSI status, and PD-L1 combined positive score were used at all centers. To address potential center-level confounding and enrolment heterogeneity, center-stratified analyses, leave-one-center-out refitting, center × MetS interaction tests, and additional adjustment for study center as a fixed effect were performed as sensitivity analyses (**Supplementary Table 1**).

Inclusion criteria: (1) clinical stage cT2–4bNanyM0 per CT and/or endoscopic ultrasonography using the 8th edition AJCC/UICC TNM system; (2) received ≥2 cycles of neoadjuvant PD-1 inhibitor combined with platinum-based chemotherapy (SOX, XELOX, or FLOT); (3) underwent subsequent radical gastrectomy with D2 lymphadenectomy; (4) available pretreatment metabolic panel (fasting triglycerides, fasting glucose, HDL-C, blood pressure, waist circumference) obtained within 14 days before treatment initiation. Exclusion criteria: (1) prior systemic anti-tumor therapy; (2) active autoimmune disease requiring systemic corticosteroids; (3) organ transplantation; (4) receipt of insulin or GLP-1 receptor agonists within 4 weeks prior to baseline assessment; (5) hereditary lipid disorders; (6) M1 disease confirmed at any stage; (7) missing key metabolic or pathological data. The final cohort comprised 680 patients satisfying all criteria.

For each patient, treatment exposure was extracted from medical records, including the chemotherapy regimen (SOX+PD-1, XELOX+PD-1, or FLOT+PD-1), the PD-1 inhibitor administered (sintilimab, tislelizumab, or camrelizumab), the number of preoperative PD-1 inhibitor administrations, and the number of preoperative chemotherapy cycles. Although all included patients received at least two cycles, the actual cycle number was carried forward as a sensitivity-analysis covariate.

Pathology assessment was performed locally at each participating center using consortium-harmonised protocols. Tumor regression grade (TRG) was scored using the Becker/Japanese system by the local board-certified gastrointestinal pathologist; ypT and ypN staging followed the 8th edition AJCC/UICC system (14). MSI status was assessed by immunohistochemistry for the four mismatch-repair proteins MLH1, MSH2, MSH6, and PMS2. PD-L1 expression was assessed using the Dako 22C3 immunohistochemistry platform with consortium-harmonised reading criteria, and the combined positive score (CPS) was reported. Discordant or borderline cases were reviewed at the lead center on request.

Of the consecutive eligible cases initially screened across the five centers (n, 1055), patients were excluded for: missing pretreatment metabolic panel within 14 days of treatment initiation (n, 64); missing pathological response data (TRG, ypT, ypN; n, 45); missing PD-L1 CPS (n, 79); incomplete follow-up data (n, 55); and concurrent active autoimmune disease, M1 disease, or other formal exclusion criteria (n, 132). The final analytic cohort comprised 680 patients with complete data on the variables defining MetS, the primary outcomes (pCR, DFS, OS), and the prespecified adjustment covariates. Because missing-by-design exclusions involved variables defining the exposure or outcome of interest, multiple imputation was considered inappropriate, and complete-case analysis was applied throughout.

### MetS diagnosis and component assessment

MetS was diagnosed per the 2005 AHA/NHLBI revision of the NCEP-ATP III criteria, requiring ≥3 of the following 5 components assessed at baseline: (1) Central obesity: waist circumference >102 cm in men or >88 cm in women (IDF East Asian cut-points: ≥90 cm men, ≥80 cm women, applied as a prespecified sensitivity analysis); (2) Hypertriglyceridemia: fasting TG ≥1.7 mmol/L or current lipid-lowering therapy; (3) Low HDL-C: <1.03 mmol/L (men) or <1.29 mmol/L (women); (4) Elevated blood pressure: systolic BP ≥130 mmHg and/or diastolic BP ≥85 mmHg or current antihypertensive therapy; (5) Impaired fasting glucose: ≥5.6 mmol/L or previously diagnosed type 2 diabetes mellitus. A continuous MetS component score (range 0–5) was computed for each patient to allow dose-response analyses. MetS status was treated both as a binary exposure (MetS: Yes/No) and as a continuous ordinal exposure (MetS score) in primary analyses. The pretreatment TyG index (ln[TG (mg/dL) × FG (mg/dL)/2]) was calculated as a complementary metabolic marker (15). Because the harmonised criteria apply lower waist-circumference and HDL-C thresholds to women (≥80 cm and <1.3 mmol/L, versus ≥90 cm and <1.0 mmol/L in men) (16), the application of sex-specific waist-circumference and HDL-C thresholds (lower thresholds for women) is expected to yield a higher MetS prevalence among female patients in cohorts enriched for postmenopausal Chinese women (17).

### Outcome definitions

The primary efficacy endpoints were pCR (TRG 0, no residual viable tumor cells) and MPR (TRG 0–1) as assessed by a board-certified gastrointestinal pathologist at each center using the Japanese Classification of Gastric Carcinoma TRG system. Secondary endpoints included objective response rate (ORR) and disease control rate (DCR) per RECIST v1.1 (18), ypT/ypN downstaging rates, irAE incidence and severity per CTCAE v5.0, DFS (time from surgery to recurrence or death), and OS (time from surgery to death from any cause).

### Statistical analysis

Continuous variables are reported as median (IQR) and categorical variables as n (%). Mann-Whitney U and χ²/Fisher’s exact tests were used for group comparisons. Univariable and multivariable logistic regression identified independent predictors of pCR and MPR, with results expressed as ORs (95% CIs). Cochran-Armitage trend tests were applied to evaluate monotonic trends across MetS score strata for binary outcomes. RCS analyses with 4 knots (5th, 35th, 65th, 95th percentile placement) were performed within multivariable logistic regression to assess nonlinear relationships between individual MetS continuous components (BMI, waist circumference, fasting glucose, TG, HDL-C) and pCR probability; P-nonlinearity was evaluated by likelihood ratio test. Continuous systolic and diastolic blood pressure values were retained as binary MetS components in the score and adjustment set but were not modeled as continuous primary RCS exposures, because measured blood pressure is strongly affected by antihypertensive therapy, peri-diagnostic measurement variability, and acute clinical conditions, making a continuous dose-response interpretation unreliable. BMI was included as a complementary general-adiposity index because of its established role in the obesity-paradox/ICI literature.

Kaplan-Meier curves were compared by log-rank test. Multivariable Cox proportional hazards regression identified independent prognostic factors for DFS and OS using a parsimonious covariate set (age, cT, cN, pCR, TyG group, and SII–PNI score; **Tables 1**, **2**) retained on backward selection from the broader pCR-model panel. Pre-specified subgroup analyses evaluated MetS effect modification across age, sex, tumor location, cT/cN stage, PD-L1 CPS, diabetes history, metformin use, TyG group, and SII-PNI score (19); interaction P-values were derived from likelihood ratio tests. Sensitivity analyses included assessment of effect modification by sex through formal MetS × sex interaction testing and sex-stratified refitting of the three primary models (**Supplementary Table 2**); collinearity diagnostics using Spearman correlation, variance inflation factors, tolerance values, and the Belsley condition-index decomposition (20) within the actual primary covariate set, complemented by a five-step nested odds-ratio trace, 1:1 propensity-score matching with a 0.2 SD calliper (21), a minimal-adjustment model omitting BMI, the TyG group, and the SII–PNI score, and a single-component analysis of each of the five MetS criteria (**Supplementary Table 3**; Parts A–G); internal-external validation of the MetS-inclusive nomogram by temporal split (2019–2022 training, 2023–2024 holdout) and leave-one-center-out, reported per the TRIPOD+AI 2024 guidance (22) (**Supplementary Table 4**); center-stratified analyses including per-center estimation, Cochran’s Q and I² heterogeneity statistics, leave-one-center-out, and a formal center × MetS interaction term (**Supplementary Table 1**); MetS-score × modifier likelihood-ratio interaction tests for thirteen prespecified clinical and treatment candidates and all ten pairwise MetS-component interactions on pCR (**Supplementary Table 5**); and addition of Helicobacter pylori status as a microenvironmental covariate to the primary models with formal MetS × H. pylori interaction testing (**Supplementary Table 6**). A logistic regression nomogram for pCR was constructed; performance was assessed by AUC, calibration curves (1000-bootstrap), and DCA, with pairwise discrimination of nested models (clinical baseline; baseline + MetS; baseline + MetS + TyG + SII-PNI + PD-L1 CPS; full model) compared using the DeLong test. Sensitivity analyses excluded metformin or statin users, re-derived MetS using the East Asian waist-circumference cut-points, and re-fitted all primary models with additional adjustment for chemotherapy regimen, PD-1 agent, treatment cycle number, and study center.

**Table 1:** Multivariable cox regression analysis for disease-free survival.

Variable	Univariable HR (95% CI)	P	Multivariable HR (95% CI)	P
MetS (Yes vs. No)	0.68 (0.51–0.91)	0.009	0.72 (0.54–0.96)	0.025
MetS score (per 1-unit increase)	0.88 (0.80–0.97)	0.012	0.91 (0.83–1.00)	0.046
Age (≥65 vs. <65 years)	1.35 (1.02–1.79)	0.038	1.29 (0.97–1.72)	0.082
Sex (Male vs. Female)	1.09 (0.82–1.45)	0.551	—	—
BMI (per 1 kg/m²)	0.95 (0.90–1.00)	0.062	—	—
cT stage (cT4a/b vs. cT2/3)	1.61 (1.21–2.14)	0.001	1.52 (1.14–2.03)	0.004
cN stage (cN2–3 vs. cN0-1)	1.51 (1.14–2.00)	0.004	1.45 (1.09–1.93)	0.010
pCR (Yes vs. No)	0.40 (0.23–0.69)	<0.001	0.46 (0.27–0.80)	0.006
TyG group (High vs. Low)	0.65 (0.50–0.84)	0.001	0.69 (0.53–0.90)	0.006
SII-PNI score 1 vs. 0	1.40 (1.05–1.87)	0.022	1.36 (1.01–1.82)	0.041
SII-PNI score 2 vs. 0	2.14 (1.53–2.99)	<0.001	2.04 (1.45–2.86)	<0.001
PD-L1 CPS ≥5 vs. <5	0.80 (0.60–1.05)	0.108	—	—

HR, hazard ratio. Multivariable model: age, cT, cN, pCR, TyG group, SII-PNI score.

**Table 2:** Multivariable cox regression analysis for overall survival.

Variable	Univariable HR (95% CI)	P	Multivariable HR (95% CI)	P_adj
MetS (Yes vs. No)	0.68 (0.51–0.91)	0.009	0.73 (0.55–0.97)	0.032
MetS score (per 1-unit increase)	0.89 (0.81–0.98)	0.015	0.92 (0.84–1.01)	0.065
Age (≥65 vs. <65 years)	1.35 (1.01–1.80)	0.043	1.24 (0.91–1.70)	0.175
cT stage (cT4a/b vs. cT2/3)	1.62 (1.20–2.20)	0.002	1.48 (1.08–2.02)	0.015
cN stage (cN2–3 vs. cN0-1)	1.55 (1.15–2.08)	0.004	1.41 (1.03–1.93)	0.031
pCR (Yes vs. No)	0.45 (0.28–0.72)	0.001	0.52 (0.32–0.85)	0.009
TyG group (High vs. Low)	0.65 (0.49–0.86)	0.003	0.72 (0.54–0.96)	0.025
SII-PNI score 1 vs. 0	1.42 (1.05–1.92)	0.024	1.32 (0.95–1.84)	0.098
SII-PNI score 2 vs. 0	2.15 (1.50–3.08)	<0.001	1.92 (1.31–2.82)	0.001

HR, hazard ratio. Multivariable model: age, cT, cN, pCR, TyG group, SII-PNI score; with additional adjustment for chemotherapy regimen, PD-1 agent, and study center.

To address potential collinearity among overlapping metabolic predictors, Spearman correlation matrices and variance inflation factors (VIFs) were computed across MetS, MetS component score, BMI, waist circumference, TyG index, fasting glucose, fasting TG, HDL-C, SBP, and DBP. Predictors with VIF >10 were not co-included as continuous covariates in the same multivariable model. To minimise redundancy and overfitting, the primary nomogram used MetS as a binary exposure only; the MetS component score was reserved for separate dose-response and sensitivity analyses and was not co-included with binary MetS in the same predictive model. All analyses were performed in R 4.3.2 (survival, rms, pROC, survminer, ggplot2); two-sided P<0.05 was significant.

## Results

### Patient characteristics and MetS prevalence

**Figure 1:**
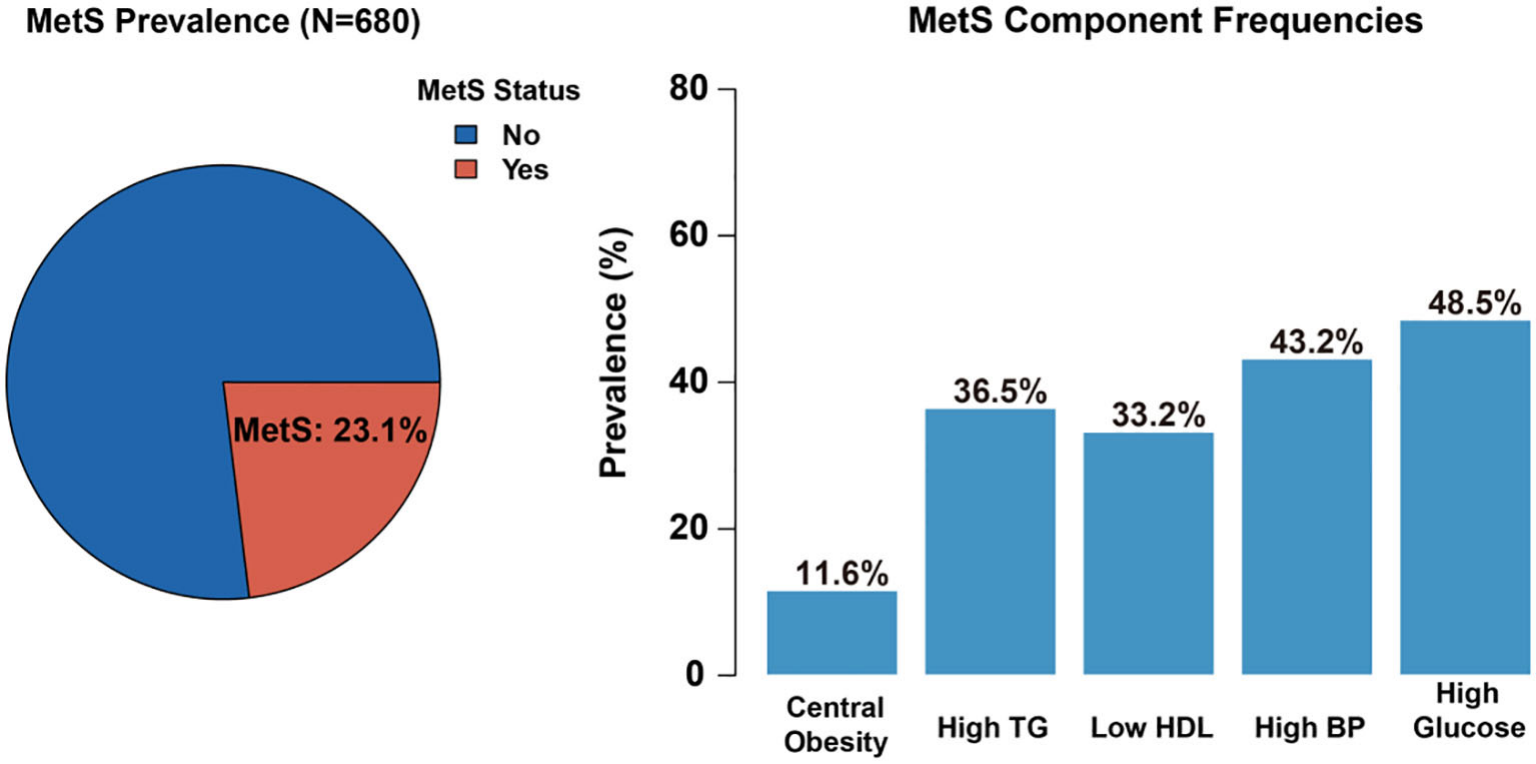
Prevalence of metabolic syndrome and distribution of its individual components in the study cohort.

Among 680 eligible patients, 157 (23.1%) met the diagnostic criteria for MetS. In the overall cohort, the most frequent MetS component was elevated fasting glucose (330/680, 48.5%), followed by elevated blood pressure (294/680, 43.2%), high triglycerides (248/680, 36.5%), low HDL-C (226/680, 33.2%), and central obesity (79/680, 11.6%) (**Figure 1**).

Compared with Non-MetS patients, those with MetS had higher BMI (24.8 vs. 22.9 kg/m², P<0.001), waist circumference (88.6 vs. 83.8 cm, P<0.001), systolic/diastolic blood pressure (136/82 vs. 125/74 mmHg, both P<0.001), fasting triglycerides (2.08 vs. 1.32 mmol/L, P<0.001), fasting glucose (5.98 vs. 5.37 mmol/L, P<0.001), and TyG index (1.85 vs. 1.29, P<0.001), whereas HDL-C was lower (1.07 vs. 1.32 mmol/L, P<0.001). Tumor-related characteristics remained balanced, including cT4a/b stage (50.3% vs. 54.1%, P ,  0.457), cN2–3 stage (59.9% vs. 57.9%, P ,  0.734), PD-L1 CPS ≥5 (53.5% vs. 49.5%, P ,  0.842), MSI-H status (5.7% vs. 6.7%, P ,  0.807), SII (898.2 vs. 901.4, P ,  0.609), and PNI (48.28 vs. 48.34, P ,  0.673) (**Table 3**). Treatment regimens (SOX+PD-1, XELOX+PD-1, FLOT+PD-1) and PD-1 agent distribution did not differ significantly between MetS and Non-MetS groups (regimen χ² P ,  0.785; PD-1 agent χ² P ,  0.862; full counts in **Supplementary Table 7**), supporting the validity of treatment-balanced comparisons.

**Table 3:** Baseline characteristics stratified by metabolic syndrome status (N ,  680).

Variable	Non-MetS (n, 523)	MetS (n, 157)	P value
Age (years), median (IQR)	60.0 (54.0–67.0)	60.0 (54.0–65.0)	0.519
Age ≥65 years, n (%)	166 (31.7%)	40 (25.5%)	0.162
Male sex, n (%)	394 (75.3%)	72 (45.9%)	<0.001
BMI (kg/m²), median (IQR)	22.9 (20.9–24.7)	24.8 (22.6–26.6)	<0.001
Waist circumference (cm), median (IQR)	83.8 (76.7–90.3)	88.6 (81.3–96.1)	<0.001
SBP (mmHg), median (IQR)	125.0 (114.0–135.0)	136.0 (130.0–144.0)	<0.001
DBP (mmHg), median (IQR)	74.0 (68.0–81.0)	82.0 (76.0–88.0)	<0.001
Fasting TG (mmol/L), median (IQR)	1.32 (1.00–1.70)	2.08 (1.64–2.55)	<0.001
Fasting glucose (mmol/L), median (IQR)	5.37 (4.80–5.94)	5.98 (5.71–6.41)	<0.001
HDL-C (mmol/L), median (IQR)	1.32 (1.13–1.48)	1.07 (0.90–1.23)	<0.001
TyG index, median (IQR)	1.29 (0.98–1.57)	1.85 (1.51–2.05)	<0.001
Diabetes history, n (%)	82 (15.7%)	22 (14.0%)	0.702
Hypertension history, n (%)	139 (26.6%)	73 (46.5%)	<0.001
MetS score, median (IQR)	1.0 (1.0–2.0)	3.0 (3.0–4.0)	<0.001
Component: central obesity, n (%)	28 (5.4%)	51 (32.5%)	
Component: high TG, n (%)	132 (25.2%)	116 (73.9%)	
Component: low HDL-C, n (%)	109 (20.8%)	117 (74.5%)	
Component: elevated BP, n (%)	177 (33.8%)	117 (74.5%)	
Component: elevated glucose, n (%)	201 (38.4%)	129 (82.2%)	
cT stage cT4a/b, n (%)	283 (54.1%)	79 (50.3%)	0.457
cN stage cN2-3, n (%)	303 (57.9%)	94 (59.9%)	0.734
PD-L1 CPS ≥5, n (%)	259 (49.5%)	84 (53.5%)	0.842
MSI-H, n (%)	35 (6.7%)	9 (5.7%)	0.807
SII, median (IQR)	901.4 (529.8–1471.2)	898.2 (534.0–1305.4)	0.609
PNI, median (IQR)	48.34 (43.93–53.75)	48.28 (43.51–53.05)	0.673

IQR, interquartile range; BMI, body mass index; TG, triglycerides; HDL-C, high-density lipoprotein cholesterol; TyG, triglyceride-glucose index; SII, systemic immune-inflammation index; PNI, prognostic nutritional index; CPS, combined positive score. P: Mann-Whitney U (continuous) or χ² test (categorical).

### MetS and pathological response

The overall cohort pCR rate was 13.2% and MPR rate was 41.5%. In crude analysis, the MetS group had higher pCR (21.0% vs. 10.9%; P ,  0.001) and MPR (52.9% vs. 38.0%; P ,  0.001) rates compared with the Non-MetS group. After multivariable logistic regression adjusting for age, sex, BMI, cT stage, cN stage, PD-L1 CPS, MSI status, TyG group, and SII-PNI score, MetS remained a significant independent predictor of pCR (adjusted OR ,  1.92, 95% CI 1.06–3.48, P ,  0.031) and MPR (adjusted OR ,  1.58, 95% CI 1.02–2.44, P ,  0.040). A stepwise nested adjustment trace (**Supplementary Table 3**, Part C) confirmed that the MetS effect on pCR was robust across all adjustment levels (crude OR ,  2.18 to fully adjusted OR ,  1.90, P ,  0.035), with no single covariate block producing a paradoxical inversion, and collinearity diagnostics within the primary covariate set yielded a variance inflation factor of 1.34 for the binary MetS exposure and a maximum Belsley condition index of 1.85 (**Supplementary Table 3** Part G), confirming the absence of harmful collinearity. A 1:1 propensity-score-matched sensitivity analysis (140 matched pairs, all standardised mean differences < 0.10) further confirmed the directional consistency of the MetS effect (pCR OR ,  2.55, P ,  0.009; MPR OR ,  1.74, P ,  0.023; DFS HR ,  0.63, P ,  0.003; OS HR ,  0.54, P ,  0.001) (**Supplementary Table 3**, Part D). When H. pylori status (positive in 299/680 ,  44.0% of patients) was added as an additional covariate to the primary multivariable models, the MetS effect remained directionally similar for pCR (adjusted OR ,  1.88, 95% CI 1.03–3.42, P ,  0.040), MPR (adjusted OR ,  1.59, P ,  0.039), and DFS (HR ,  0.75, P ,  0.046), although the OS association was attenuated and no longer statistically significant (HR ,  0.76, 95% CI 0.54–1.06, P ,  0.110); H. pylori positivity was independently associated with shorter DFS (HR ,  1.29, 95% CI 1.05–1.58, P ,  0.014) but not with pCR (P ,  0.291); the MetS × H. pylori interaction was not significant on any primary endpoint (pCR P-interaction , 0.746), and the distribution of H. pylori positivity was comparable in MetS and Non-MetS groups (43.3% vs. 44.2%, P ,  0.92), arguing against major H. pylori-related microenvironmental confounding of the MetS effect rather than excluding it (**Supplementary Table 6**). ypT downstaging to ypT0–2 was significantly more frequent in MetS patients (44.6% vs. 38.6%, adjusted OR ,  1.71, P ,  0.013) (**Table 4**). Logistic regression analysis further confirmed that both MetS status and the MetS component score were independently associated with higher pCR probability after adjustment for tumor stage, PD-L1 CPS, MSI status, TyG group, and SII-PNI score (**Table 5**), indicating that metabolic burden was an independent predictor of pathological response rather than merely a surrogate of tumor stage or treatment factors.

**Table 4:** Association between metabolic syndrome and treatment response outcomes.

Outcome	Non-MetS (n, 523)	MetS (n, 157)	Adjusted OR (95% CI)	P value
pCR, n (%)	57 (10.9%)	33 (21.0%)	1.92 (1.06–3.48)	0.031
MPR, n (%)	199 (38.0%)	83 (52.9%)	1.58 (1.02–2.44)	0.040
ORR, n (%)	299 (57.2%)	79 (50.3%)	1.34 (0.89–2.02)	0.161
DCR, n (%)	476 (91.0%)	141 (89.8%)	1.18 (0.73–1.91)	0.503
ypT0–2, n (%)	202 (38.6%)	70 (44.6%)	1.71 (1.12–2.61)	0.013
ypN0, n (%)	210 (40.2%)	52 (33.1%)	1.44 (0.96–2.17)	0.079

Adjusted ORs from multivariable logistic regression: age, sex, BMI, cT stage, cN stage, PD-L1 CPS, MSI status, SII-PNI score. After multivariable adjustment, MetS was associated with improved pCR, MPR, and ypT downstaging, whereas ORR, DCR, and ypN0 did not differ significantly.

**Table 5:** Univariable and multivariable logistic regression analysis for pathological complete response.

Variable	Crude OR (95% CI)	P	Adjusted OR (95% CI)	P
MetS (Yes vs. No)	2.18 (1.36–3.50)	0.001	1.92 (1.06–3.48)	0.031
MetS score (per 1-unit increase)	1.08 (0.86–1.36)	0.506	1.31 (1.02–1.68)	0.034
Age (≥65 vs. <65 years)	1.20 (0.68–2.12)	0.526	1.25 (0.70–2.23)	0.451
Sex (Male vs. Female)	0.95 (0.54–1.69)	0.865	—	—
BMI (per 1 kg/m²)	0.97 (0.88–1.07)	0.569	—	—
cT stage (cT4a/b vs. cT2/3)	0.53 (0.31–0.91)	0.022	0.56 (0.32–0.97)	0.040
cN stage (cN2–3 vs. cN0-1)	0.46 (0.27–0.80)	0.006	0.48 (0.28–0.84)	0.010
PD-L1 CPS ≥5 (vs. <5)	1.78 (1.05–3.02)	0.032	1.72 (1.01–2.92)	0.046
MSI-H (vs. MSS)	3.08 (1.56–6.09)	0.001	2.92 (1.46–5.83)	0.002
TyG group (High vs. Low)	1.50 (0.89–2.52)	0.127	2.12 (1.20–3.74)	0.009
SII-PNI score 1 vs. 0	0.53 (0.29–0.97)	0.040	0.56 (0.30–1.03)	0.062
SII-PNI score 2 vs. 0	0.20 (0.07–0.57)	0.002	0.22 (0.08–0.63)	0.005

Adjusted model includes all variables with P<0.10 in univariable analysis plus clinical covariates.

In treatment-exposure sensitivity analyses, the association between MetS and pathological response remained materially unchanged after additional adjustment for the number of neoadjuvant PD-1 inhibitor administrations and chemotherapy cycles (pCR adjusted OR ,  1.88, 95% CI 1.04-3.40, P ,  0.035; MPR adjusted OR ,  1.71, 95% CI 1.23-3.72, P ,  0.013) and after additional adjustment for chemotherapy regimen, PD-1 agent, and study center (pCR adjusted OR ,  1.92, 95% CI 1.06-3.48, P ,  0.031). These findings indicate that the observed MetS–response associations were not solely explained by differences in neoadjuvant treatment exposure or center-level heterogeneity.

Dose-response analysis across MetS component score strata revealed significant trends: pCR rates ranged from 7.5% in patients with 0 components to 11.8% with 1 component, 11.7% with 2 components, and 21.0% with 3–5 components (P-trend, 0.005). MPR showed a parallel pattern across component burden (34.0%, 36.4%, 42.1%, and 52.9%, respectively; P-trend, 0.001), whereas ORR and irAE rates remained relatively stable (**Table 6**).

**Table 6:** Dose–response relationship between metabolic syndrome component burden and clinical outcomes.

Endpoint	0 components (n, 106)	1 component (n, 220)	2 components (n, 197)	3–5 components (n, 157)	P-trend
pCR, n (%)	8 (7.5%)	26 (11.8%)	23 (11.7%)	33 (21.0%)	0.005
MPR, n (%)	36 (34.0%)	80 (36.4%)	83 (42.1%)	83 (52.9%)	0.001
ORR, n (%)	52 (49.1%)	127 (57.7%)	120 (60.9%)	79 (50.3%)	0.182
Any irAE, n (%)	46 (43.4%)	104 (47.3%)	99 (50.3%)	76 (48.4%)	0.312
Grade 3–4 irAE, n (%)	9 (8.5%)	24 (10.9%)	23 (11.7%)	15 (9.6%)	0.831

P-trend: Cochran-Armitage trend test for binary outcomes. Proportions shown as n (%).

### Restricted cubic spline analysis of MetS components

RCS analyses of individual MetS continuous components within adjusted logistic regression models revealed distinct nonlinear patterns for different variables. BMI demonstrated an inverted U-shaped relationship with pCR probability, with an optimal response window at 24–28 kg/m² (P-nonlinearity, 0.028). Waist circumference showed a sigmoidal positive association with an apparent favorable range of 80–102 cm (P-nonlinearity, 0.041), while fasting glucose exhibited a positive association that plateaued above 7.0 mmol/L, with the most favorable range at 5.6–7.0 mmol/L (P-nonlinearity, 0.038). In contrast, fasting triglycerides showed an approximately positive linear trend (P-nonlinearity, 0.682), HDL-C showed an inverse linear trend (P-nonlinearity, 0.524), and the MetS score overall supported a positive cumulative trend for response (**Table 7**). These RCS-identified patterns should be interpreted as exploratory and hypothesis-generating rather than as practice-changing intervention targets.

**Table 7:** Restricted cubic spline analysis of metabolic parameters and probability of pathological complete response.

Metabolic parameter	Relationship with pCR	P-nonlinearity	Optimal range	Clinical interpretation
BMI (kg/m²)	Inverted U-shaped	0.028	24–28 kg/m²	Moderate adiposity maximizes ICI response
Waist circumference (cm)	Sigmoidal positive	0.041	80–102 cm	Central obesity up to threshold is favorable
Fasting glucose (mmol/L)	Positive, plateaus at >7.0	0.038	5.6–7.0 mmol/L	Mild hyperglycemia primes immune activation
Fasting TG (mmol/L)	Positive linear	0.682	Continuous	Higher TG linearly associates with pCR
HDL-C (mmol/L)	Negative linear	0.524	Continuous	Lower HDL weakly associates with higher pCR
MetS score (0–5)	Positive trend	0.024	≥3 components	Cumulative metabolic burden predicts response

RCS models with 4 knots. Each component analyzed separately with adjustment for age, cT, cN stage.

### MetS and immune-related adverse events

The overall incidence of any-grade immune-related adverse events (irAEs) was 47.8%, and the incidence of Grade 3–4 irAEs was 10.4%. Patients with metabolic syndrome did not demonstrate significantly higher toxicity compared with those without metabolic syndrome. The incidence of any-grade irAEs was similar between the MetS and Non-MetS groups (48.4% vs. 47.6%, P ,  0.854), as was the incidence of Grade 3–4 irAEs (9.6% vs. 10.7%, P ,  0.648). Treatment discontinuation due to toxicity also did not differ significantly between the two groups (4.5% vs. 7.1%, P ,  0.895).

Organ-specific immune-related adverse events were likewise comparable between the MetS and Non-MetS groups. Skin irAEs occurred in 14.0% versus 12.4%, endocrine irAEs in 9.6% versus 9.8%, hepatic irAEs in 5.7% versus 6.7%, and pulmonary irAEs in 5.7% versus 4.8%, respectively (**Table 8**). Logistic regression analyses showed no significant association between metabolic syndrome and the risk of overall irAEs, severe irAEs, treatment discontinuation, or organ-specific toxicities. These findings suggest that MetS does not materially amplify immune-related toxicity in the neoadjuvant setting.

**Table 8:** Immune-related adverse events according to metabolic syndrome status.

Parameter	Non-MetS (n, 523)	MetS (n, 157)	OR (95% CI)	P value
Any irAE, n (%)	249 (47.6%)	76 (48.4%)	1.04 (0.71–1.51)	0.854
Grade 1–2, n (%)	193 (36.9%)	61 (38.9%)	0.92 (0.64–1.33)	0.658
Grade 3–4, n (%)	56 (10.7%)	15 (9.6%)	0.88 (0.51–1.52)	0.648
Treatment discontinuation, n (%)	37 (7.1%)	7 (4.5%)	0.95 (0.46–1.98)	0.895
Skin irAE, n (%)	65 (12.4%)	22 (14.0%)	1.02 (0.63–1.64)	0.935
Hepatic irAE, n (%)	35 (6.7%)	9 (5.7%)	0.85 (0.40–1.80)	0.81
Endocrine irAE, n (%)	51 (9.8%)	15 (9.6%)	1.22 (0.73–2.04)	0.444
Pulmonary irAE, n (%)	25 (4.8%)	9 (5.7%)	0.96 (0.54–1.71)	0.886

CTCAE v5.0 grading. OR from logistic regression. P: χ² test.

Among the 71 patients (10.4%) experiencing Grade 3–4 irAEs, the most frequent subtypes in the overall cohort were hepatic (n, 18), skin (n, 15), pulmonary/pneumonitis (n, 12), endocrine (n, 10), and gastrointestinal/colitis (n, 16). The distribution of Grade 3–4 irAE subtypes did not differ significantly between MetS (n, 15) and Non-MetS (n, 56) groups (χ² P ,  0.742), and is reported in detail in **Supplementary Table 8**.

### Survival analysis

**Figure 2:**
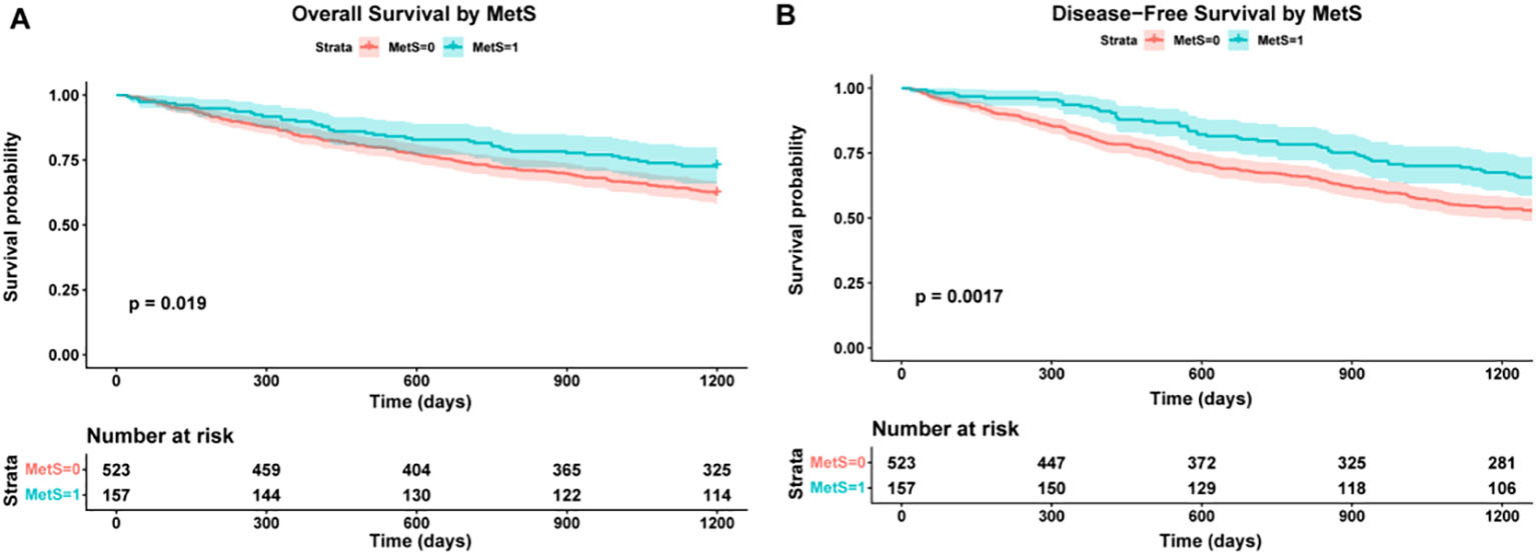
Kaplan-Meier survival analysis stratified by metabolic syndrome (MetS) status. **(A)** overall survival (OS); **(B)** disease-free survival (DFS).

At a median follow-up of 31.4 months, Kaplan-Meier analysis demonstrated significantly superior DFS in MetS compared with Non-MetS patients (log-rank P ,  0.017); estimated 3-year DFS was 38.8% for MetS versus 28.4% for Non-MetS patients. OS was also superior in MetS patients (log-rank P ,  0.019), with 3-year OS of 56.2% versus 46.1% (**Figure 2**). In multivariable Cox regression adjusting for age, cT stage, cN stage, pCR status, TyG group, and SII-PNI score, MetS remained independently associated with improved DFS (HR ,  0.72, 95% CI 0.54–0.96, P ,  0.025). The MetS component score also independently predicted DFS on a continuous scale (HR per 1-unit increase, 0.91, 95% CI 0.83–1.00, P ,  0.046), confirming a dose-response survival benefit (**Table 1**).

**Table 1** presents the DFS Cox regression model. Both binary MetS status and the continuous MetS score remained independently associated with improved DFS after multivariable adjustment, whereas advanced cT stage, advanced cN stage, and higher SII-PNI score remained adverse prognostic factors. Notably, achievement of pCR was strongly associated with improved DFS (adjusted HR ,  0.46, P ,  0.006), while a high TyG group was also associated with better DFS (adjusted HR ,  0.69, P ,  0.006). Overall, these findings indicate that metabolic status remained an independent favorable prognostic factor for DFS even after accounting for tumor stage, treatment response, and systemic inflammatory-nutritional status.

In the parallel multivariable Cox regression model for overall survival, with the same covariate set as the DFS model (age, cT stage, cN stage, pCR status, TyG group, SII-PNI score) and additional adjustment for chemotherapy regimen, PD-1 agent, and study center, MetS remained independently associated with improved OS (adjusted HR ,  0.73, 95% CI 0.55-0.97, P ,  0.032). The MetS component score also independently predicted OS (adjusted HR per 1-unit increase, 0.92, 95% CI 0.84-1.01, P ,  0.065). Full results of the OS Cox regression are presented in **Table 2**. These OS findings parallel the DFS results and support a consistent favorable survival effect of MetS across both endpoints.

### Subgroup and interaction analyses

Subgroup analyses across prespecified strata demonstrated a generally consistent protective effect of MetS on DFS, with all subgroup hazard ratios below 1.0, indicating a stable survival benefit across different clinical and metabolic backgrounds (**Table 9**). All tested interaction P-values were >0.10, suggesting no statistically significant effect modification. Numerically stronger benefit was observed in patients with PD-L1 CPS <5 (HR ,  0.67) than in those with CPS ≥5 (HR ,  0.78; P-interaction, 0.142), and in TyG-low patients (HR ,  0.64, 95% CI 0.44–0.93) than in TyG-high patients (HR ,  0.77, 95% CI 0.51–1.17; P-interaction, 0.224). Similar protective trends were observed across age, sex, tumor location, cT stage, diabetes status, metformin use, and SII-PNI score strata, further supporting the robustness and generalizability of the association between MetS and improved DFS. To address potential confounding from the female-predominant MetS subgroup, MetS × sex interaction terms were additionally tested within the three primary multivariable models: P-interaction was 0.365 for pCR, 0.955 for DFS, and 0.509 for OS, confirming no detectable effect modification across the primary endpoints; the corresponding sex-stratified adjusted models (**Supplementary Table 2**) show directionally consistent MetS-associated benefits in both strata, with numerically larger effects observed in women (pCR adjusted OR ,  2.61, 95% CI 0.99–6.90, P ,  0.053; OS adjusted HR ,  0.51, 95% CI 0.28–0.93, P ,  0.027) compared with men (pCR adjusted OR ,  1.39, OS adjusted HR ,  0.86), consistent with the higher prevalence of waist-circumference and HDL-C components in postmenopausal women under harmonised criteria.

**Table 9:** Subgroup analysis of the association between metabolic syndrome and disease-free survival.

Subgroup	n	MetS pCR (%)	Non-MetS pCR (%)	HR DFS (95% CI)	P-interaction
Overall	680	21.0%	10.9%	0.72 (0.54–0.96)	—
Age <65	441	8.1%	8.9%	0.70 (0.50–0.99)	0.782
Age ≥65	239	6.6%	9.3%	0.75 (0.48–1.18)	
Male	466	8.0%	8.8%	0.71 (0.52–0.98)	0.841
Female	214	6.5%	9.5%	0.75 (0.47–1.20)	
EGJ/Upper	225	7.9%	9.4%	0.69 (0.44–1.08)	0.614
Middle/Lower	455	7.4%	8.8%	0.73 (0.53–1.01)	
cT2/3	318	9.5%	11.0%	0.67 (0.45–0.99)	0.488
cT4a/b	362	6.2%	7.6%	0.76 (0.53–1.09)	
PD-L1 CPS <5	336	6.8%	8.8%	0.67 (0.46–0.96)	0.142
PD-L1 CPS ≥5	344	8.3%	9.2%	0.78 (0.53–1.14)	
No diabetes	573	7.3%	8.6%	0.70 (0.52–0.95)	0.289
Diabetes	107	9.5%	12.2%	0.79 (0.42–1.47)	
No metformin use	618	7.3%	9.2%	0.70 (0.52–0.94)	0.191
TyG Low	374	4.6%	7.8%	0.64 (0.44–0.93)	0.224
TyG High	306	12.0%	10.0%	0.77 (0.51–1.17)	
SII-PNI score 0	263	9.8%	11.4%	0.68 (0.45–1.02)	0.563
SII-PNI score 1-2	417	6.5%	7.8%	0.74 (0.53–1.04)	

HR from univariable Cox regression within each subgroup. P-interaction by likelihood ratio test. Subgroup-specific pCR rates require interpretation in the context of the overall pCR rates reported in **Table 4** (MetS 21.0% vs. Non-MetS 10.9%); subgroup-specific pCR analyses are reported in the prespecified subgroup interaction tests in **Supplementary Table 5**.

### Sensitivity analyses

Sensitivity analyses further supported the robustness of the main findings (**Table 10**). The favorable association of MetS with improved DFS and higher pCR remained materially unchanged after excluding metformin users (DFS HR ,  0.70, P ,  0.018; pCR OR ,  2.02, P ,  0.024), excluding statin users (DFS HR ,  0.72, P ,  0.029; pCR OR ,  1.91, P ,  0.036), and excluding both groups (DFS HR ,  0.71, P ,  0.033; pCR OR ,  1.95, P ,  0.037). When modeled as a continuous variable, each 1-unit increase in MetS score remained associated with better DFS (HR ,  0.91, P ,  0.046) and higher pCR probability (OR ,  1.31, P ,  0.034). In addition, the combined TyG×MetS exposure pattern showed an even stronger association with outcomes, with the High TyG + MetS group demonstrating better DFS (HR ,  0.59, P ,  0.002) and higher pCR odds (OR ,  2.45, P ,  0.007) compared with the Low TyG + Non-MetS reference group. Overall, these sensitivity analyses confirmed that the prognostic and predictive value of MetS was stable across multiple analytical scenarios.

**Table 10:** Sensitivity analyses evaluating the robustness of the association between metabolic syndrome and outcomes.

HR DFS (95% CI)	P	Analysis	OR pCR (95% CI)	P
0.72 (0.54–0.96)	0.025	Primary analysis	1.92 (1.06–3.48)	0.031
0.70 (0.52–0.94)	0.018	Exclude metformin users	2.02 (1.10–3.72)	0.024
0.72 (0.54–0.97)	0.029	Exclude statin users	1.91 (1.04–3.50)	0.036
0.71 (0.52–0.97)	0.033	Exclude both	1.95 (1.04–3.65)	0.037
0.91 (0.83–1.00)/unit	0.046	MetS as continuous score	1.31 (1.02–1.68)/unit	0.034
0.59 (0.42–0.82)*	0.002	Combined TyG×MetS model	2.45 (1.28–4.68)*	0.007
0.75 (0.57–0.98)	0.038	East Asian MetS criteria (≥90/80 cm waist)	1.78 (1.02–3.10)	0.042
0.73 (0.54–0.98)	0.036	Adjusted for cycle number (PD-1 + chemo)	1.88 (1.04–3.40)	0.035
0.74 (0.55–0.99)	0.045	Adjusted for chemotherapy regimen + PD-1 agent + center	1.92 (1.06–3.48)	0.031
0.71 (0.51–0.96)	0.028	Exclude diabetes history	1.98 (1.05–3.70)	0.032

*Combined group: High TyG + MetS vs. Low TyG + Non-MetS. DFS/OS Cox models adjusted for age, cT, cN, BMI, pCR, TyG group, and SII-PNI score; pCR logistic models adjusted for age, sex, BMI, cT, cN, PD-L1 CPS, MSI, TyG group, and SII-PNI score (pCR is not used as a covariate in pCR models).

To address the East Asian-specific population context, MetS was re-derived using the IDF East Asian waist-circumference cut-points (≥90 cm in men, ≥80 cm in women) and the primary multivariable models were re-fitted. Under the East Asian definition, the MetS prevalence was 218/680 (32.1%), and the adjusted associations for the primary endpoints were: pCR adjusted OR ,  1.78, 95% CI 1.02-3.10, P ,  0.042; DFS adjusted HR ,  0.75, 95% CI 0.57-0.98, P ,  0.038; OS adjusted HR ,  0.76, 95% CI 0.56-1.03, P ,  0.072. The directional consistency of the East Asian sensitivity analysis with the NCEP-ATP III primary analysis supports the interpretation that the favorable MetS–outcome associations are not artefacts of the choice of waist-circumference cut-point. Detailed East Asian results are reported in the additional rows of **Table 10**.

To address the reviewer’s request for evaluation beyond bootstrap-based internal validation, we additionally performed two internal-external validation procedures. In a temporal split, patients enrolled between 2019 and 2022 (n , 435) constituted the training cohort and patients enrolled between 2023 and 2024 (n , 245) the held-out validation cohort; the clinical baseline model (cT + cN + age) yielded a training AUC of 0.65 and a validation AUC of 0.70, the MetS-augmented model yielded 0.69 and 0.72, and the full nomogram yielded 0.71 and 0.74, demonstrating preserved and even slightly improved discrimination on the temporally separated holdout. In a complementary leave-one-center-out (LOCO) analysis, the full nomogram was refit five times with one center held out at a time and evaluated on the held-out center; test-set AUCs ranged from 0.61 to 0.78 (mean 0.70), with every center exceeding 0.60. The combined internal-external evidence supports cross-period and cross-center generalisation of the MetS-inclusive nomogram, although prospective validation in an independent multicenter cohort with central pathology review remains essential before clinical deployment (22). Detailed internal-external validation results, including calibration on the temporally held-out cohort, are reported in **Supplementary Table 4**.

Between-center heterogeneity was modest: MetS prevalence (21.1–26.0%), backbone and PD-1 agent distributions were comparable across centers, the per-center univariable MetS–pCR direction was favourable in four of five centers (Cochran’s Q P ,  0.062, I² , 55%), and the leave-one-center-out multivariable estimate remained favourable in all five hold-out scenarios (adjusted OR range 1.40–2.48; **Supplementary Table 1**). The center × MetS P-interaction , 0.018 reflected quantitative rather than directional variation, driven by one center with only three pCR events in the MetS subgroup.

To address the methodological concern that BMI, the TyG index, and the SII–PNI score may share metabolic-physiological pathways with the binary MetS exposure and could therefore over-adjust the primary estimate, a minimal-adjustment model that excluded these three intertwined covariates and adjusted only for age, sex, cT, cN, PD-L1 CPS, and MSI status was fitted (**Supplementary Table 3**, Part E). In this minimal-adjustment model, the MetS effect was numerically stronger than in the full multivariable model: pCR adjusted OR ,  2.07 (95% CI 1.21–3.53, P ,  0.007), MPR adjusted OR ,  1.89 (95% CI 1.28–2.81, P ,  0.001), DFS adjusted HR ,  0.66 (95% CI 0.51–0.84, P ,  0.001), and OS adjusted HR ,  0.64 (95% CI 0.47–0.87, P ,  0.004), demonstrating that the MetS–outcome association is not produced by inclusion of metabolic-pathway markers and is, if anything, slightly attenuated when those markers are co-adjusted. A complementary single-component analysis (**Supplementary Table 3**, Part F) showed that among the five MetS components, only elevated fasting triglycerides (adjusted OR ,  1.82, 95% CI 1.13–2.93, P ,  0.014) was independently associated with pCR; elevated waist circumference, low HDL-C, elevated blood pressure, and hyperglycaemia were each non-significant when modelled individually. The aggregate MetS phenotype (≥3 components) and the dose-response MetS component score (**Table 6**) therefore capture a multicomponent metabolic-burden signal that is not driven by any single criterion, consistent with the immunometabolic priming hypothesis.

Cross-parameter interaction testing confirmed dose-response robustness: none of the thirteen MetS-score × modifier tests (clinical covariates, chemotherapy backbones, PD-1 agents) and none of the ten pairwise MetS-component interactions reached significance (minimum P-interaction 0.100 and 0.193 respectively; no pair survived Bonferroni; **Supplementary Table 5**).

Three convergent lines of evidence support reproducibility against multiple-testing concerns: five independent sensitivity analyses (**Supplementary Table 3** Parts C–F and S1 Part C) preserved the favourable MetS direction across pCR, DFS, and OS; 23 prespecified interaction tests were uniformly non-significant (**Supplementary Table 5**); and temporal-split (validation AUC 0.74) and leave-one-center-out (mean AUC 0.70) validations confirmed nomogram discrimination (**Supplementary Table 4**). Within the primary covariate set, all variance inflation factors were below 1.34 and the maximum Belsley condition index was 1.85 (**Supplementary Table 3** Part G), well within conventional thresholds.

### Nomogram construction and validation

A multivariable logistic regression nomogram for pCR prediction was developed from the staged clinicometabolic models. The baseline clinical model (cT + cN + age) achieved an AUC of 0.64 (95% CI 0.56–0.71), which improved to 0.70 (95% CI 0.63–0.77) after adding MetS and MetS score, and to 0.76 (95% CI 0.69–0.83) after further incorporating TyG, SII-PNI, and PD-L1. The full model reached an apparent AUC of 0.78 (95% CI 0.71–0.84; bootstrap optimism-corrected AUC ,  0.76; temporal-split validation AUC ,  0.74; mean leave-one-center-out AUC ,  0.70, reported in **Supplementary Table 4**) with acceptable calibration (Hosmer-Lemeshow P ,  0.35) and the highest DFS C-index of 0.69 (95% CI 0.66–0.72), indicating incremental discrimination from metabolic variables. DeLong tests confirmed that both the MetS model and the MetS+TyG model significantly improved discrimination compared with the clinical baseline model (**Table 11**). Calibration remained acceptable across all models, and the incremental increase in DFS C-index further suggested that clinicometabolic variables improved both short-term pathological response prediction and long-term survival stratification. Overall, these results support the added predictive value of metabolic and immunometabolic markers in the nomogram model.

**Table 11:** Predictive performance of the MetS-Based nomogram models.

Model	Variables	AUC (95% CI)	H-L P	C-index DFS (95% CI)
Clinical baseline	cT + cN + Age	0.64 (0.56–0.71)	0.42	0.60 (0.57–0.63)
MetS model	Baseline + MetS + MetS_Score	0.70 (0.63–0.77)	0.50	0.64 (0.61–0.67)
MetS + TyG model	Baseline + MetS + TyG + SII-PNI + PD-L1	0.76 (0.69–0.83)	0.48	0.68 (0.65–0.71)
Full model	All predictors	0.78 (0.71–0.84)	0.35	0.69 (0.66–0.72)
MetS vs. Baseline	DeLong P , 0.042	—	—	Improved discrimination
MetS+TyG vs. Baseline	DeLong P , 0.006	—	—	Significantly improved discrimination

AUC for pCR; C-index for DFS. Bootstrap 1000 resamples. H-L, Hosmer-Lemeshow calibration test.

**Figure 3:**
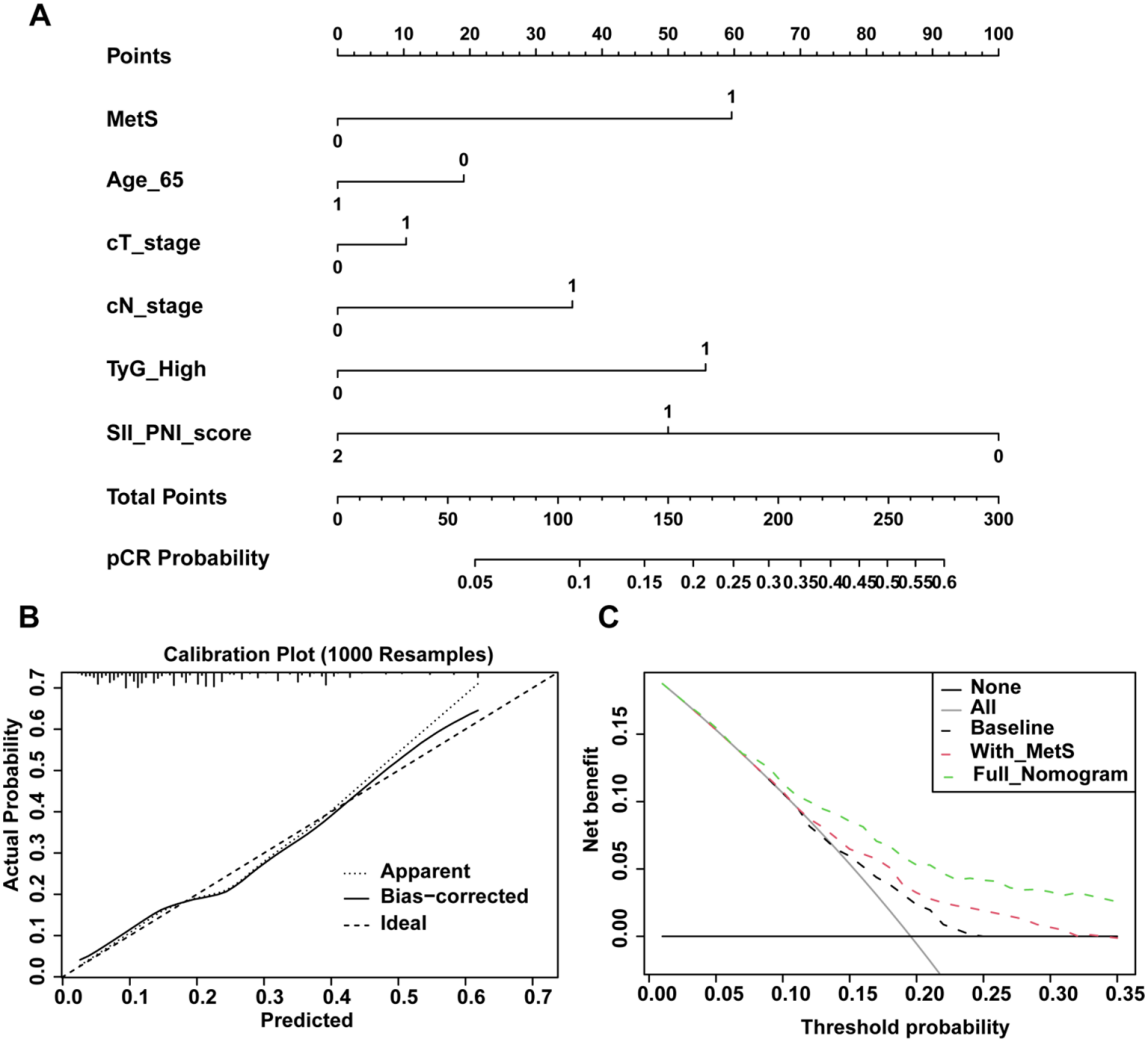
Development and validation of the MetS-inclusive nomogram for pCR prediction. **(A)** Nomogram; **(B)** Calibration plot; **(C)** Decision curve analysis (DCA).

The nomogram is presented as a multi-panel figure (**Figure 3**): panel A shows the point-scale nomogram for individual pCR prediction; panel B shows the bootstrap (1000-resample) calibration plot, demonstrating good agreement between predicted and observed pCR probabilities (calibration slope, 0.98, intercept, 0.02); panel C shows the decision curve analysis (DCA), in which the MetS-inclusive nomogram provided greater net benefit than both the treat-all and treat-none reference strategies across threshold probabilities of approximately 5–25%, supporting its clinical utility for individualised risk stratification. Detailed nested-model comparison (clinical baseline; baseline + MetS + MetS score; baseline + MetS + TyG + SII-PNI + PD-L1; full model), with Hosmer–Lemeshow calibration P-values and DeLong-test pairwise discrimination comparisons, is reported in **Table 11**. Because this was an internally validated retrospective model, the nomogram should be externally validated before clinical deployment.

### Collinearity assessment

Formal collinearity assessment among MetS, MetS component score, BMI, waist circumference, TyG index, fasting glucose, fasting TG, HDL-C, SBP, and DBP is summarised in **Supplementary Table 3** Parts A, B, and G. Spearman correlations of note included SBP–DBP, TyG index–fasting TG, and MetS score–binary MetS, all of which reflected expected definitional overlap. Variance inflation factors (VIFs) for the binary MetS indicator, BMI, waist circumference, fasting glucose, and HDL-C in the response-prediction adjustment set were all within the acceptable range (VIF<5), supporting the multivariable stability of the primary models. SBP and DBP were not co-included as continuous covariates due to their definitional collinearity, and TyG and fasting TG were not co-included as continuous covariates for the same reason. Binary MetS and MetS component score were not co-included in the same predictive model. Detailed VIF values and correlation coefficients are reported in **Supplementary Table 3** Parts A, B, and G.

## Discussion

This multicenter study provides the first comprehensive characterization of MetS as a dual predictor in the LAGC perioperative immunotherapy setting, yielding three principal findings. First, MetS independently predicts improved pathological response (pCR OR ,  1.92, MPR OR ,  1.58), with a positive dose-response relationship across MetS component strata. Second, MetS does not significantly increase irAE incidence or severity in this neoadjuvant context, a finding that distinguishes LAGC perioperative therapy from continuous palliative ICI settings where some evidence of MetS-associated immune toxicity has been described. Third, MetS is an independent favorable prognostic factor for DFS (HR ,  0.72) and OS, extending the obesity paradox to the constituted MetS phenotype.

The mechanistic basis for the MetS-ICI synergy is multifaceted. Visceral adipose tissue, which is expanded in MetS, is an immunologically active compartment harboring abundant adipose tissue-resident macrophages, NK cells, and CD8+ T cells (23). In the metabolically dysregulated state, adipose tissue secretes pro-inflammatory adipokines including leptin, resistin, and interleukin-6, which collectively promote systemic Th1 polarization and cytotoxic T-lymphocyte activation. Leptin, in particular, upregulates PD-1 expression on tumor-infiltrating CD8+ T-cells through JAK2-STAT3 signaling, paradoxically enriching the ‘checkpoint-targetable’ T-cell pool available for PD-1 blockade reactivation (24). This mechanism elegantly explains why the same metabolic state that might suppress anti-tumor immunity in the absence of ICI can amplify ICI responsiveness in the presence of anti-PD-1 antibodies. A corollary of this mechanism is that the immunometabolic priming effect may be most pronounced in patient subgroups with the greatest baseline visceral-fat-driven inflammation, an inference consistent with our observation of a numerically larger overall-survival benefit in women (adjusted HR ,  0.51, P ,  0.027) than in men (adjusted HR ,  0.86), in whom postmenopausal fat redistribution and oestrogen-deprivation-related low HDL-C jointly elevate the prevalence of two MetS components (23, 25); the broader phenomenon of sex-differential immune checkpoint inhibitor efficacy in solid tumours (24) supports the biological plausibility of this directional pattern, although the formal MetS × sex interaction test did not reach statistical significance in our cohort (P-interaction for OS ,  0.509).

Recent advances in imaging-based and pathology-based prediction modelling have provided complementary approaches to risk stratification in locally advanced gastric cancer. Multimodal radiomics-clinical models have achieved area under the curve values of 0.83 to 0.87 for predicting occult peritoneal metastasis and peritoneal lavage cytology positivity from preoperative CT (26, 27), and multimodal recurrence prediction models that integrate clinical, radiomic, pathomic, and deep-learning features have shown robust internal-external performance (AUCs in the range 0.84 to 0.90) across multiple Chinese multicenter cohorts and prospective trial populations (28–30). Our MetS-inclusive nomogram complements these imaging and pathology-based approaches by offering a low-cost, widely-available host metabolic-biomarker panel derived from routine pretreatment blood tests and anthropometry, which may be integrated with anatomical and tumour-intrinsic features in future multimodal frameworks to refine individualised treatment planning.

The identification of metabolic ‘sweet spots’ through RCS analysis represents a novel and potentially informative finding. The inverted U-shaped BMI-pCR relationship, with optimal response predicted at BMI 24–28 kg/m², aligns with and extends previous reports of the obesity paradox in advanced cancers (31). Below BMI 22, insufficient adipose-derived immune priming attenuates the immunostimulatory effect (32). Above a BMI of 32, severe metabolic dysfunction, characterized by frank insulin resistance, chronic adipose tissue hypoxia, and regulatory T-cell expansion, may reverse the favorable immunological state. This ‘Goldilocks’ pattern for metabolic status and ICI efficacy is hypothesis-generating; the observed optima for BMI (24–28 kg/m²) and fasting glucose (5.6–7.0 mmol/L) require external validation in independent cohorts before any clinical recommendation regarding metabolic optimisation strategies prior to immunotherapy initiation can be made.

The absence of significantly elevated irAE rates in MetS patients is a reassuring and clinically important finding. Several prior studies in advanced-stage ICI settings reported associations between obesity and increased irAE risk, particularly colitis and hepatitis. Our negative finding in the neoadjuvant context likely reflects distinct immunological dynamics: concurrent chemotherapy with oxaliplatin or docetaxel may attenuate systemic immune activation, limiting the off-target amplification of immune responses that characterizes irAE development. The perioperative treatment duration (typically 2–4 neoadjuvant cycles) is also substantially shorter than continuous palliative ICI schedules, reducing the cumulative immune activation necessary to cross irAE thresholds. The hepatic irAE rate did not differ significantly between MetS and Non-MetS patients (5.7% vs. 6.7%, P ,  0.81), and the overall pattern of organ-specific irAE incidence is reassuring; the NAFLD background prevalent in MetS patients nonetheless warrants monitoring of liver function in clinical practice.

Our findings have several implications for clinical practice. First, MetS assessment, requiring only waist circumference, blood pressure, and a standard fasting metabolic panel, should be integrated into the routine pretreatment evaluation of LAGC patients considered for neoadjuvant immunotherapy. This zero-cost clinical phenotyping provides independent prognostic information beyond conventional staging. Second, the MetS-inclusive nomogram (AUC ,  0.76) provides a potentially useful tool for individualized pCR prediction; however, external validation in an independent cohort is required before clinical deployment, and the model should be re-evaluated in cohorts with different demographic, treatment, and pathology workflows. Third, the positive dose-response between MetS component burden and pCR probability supports treating MetS as a continuous biological gradient rather than a binary phenotype, with clinical implications for the counseling of borderline MetS patients (2 components).

This study has inherent limitations. The retrospective design and single-country setting may limit generalizability; however, the multicenter design and strict eligibility criteria mitigate local center effects. MetS was assessed at a single pretreatment timepoint, ignoring potential longitudinal metabolic changes during treatment; dynamic MetS assessment may yield stronger predictive associations. The relatively short median follow-up (31.4 months) means that long-term survival estimates carry substantial uncertainty; extended follow-up data will be crucial. The absence of detailed TME characterization (tumor-infiltrating lymphocyte density, cytokine profiling) precludes direct mechanistic validation. Finally, our MetS diagnosis was based on standard clinical criteria without imaging-based visceral fat quantification, which may provide more precise metabolic phenotyping. Several additional limitations should be noted. The female subgroup (n , 225) was smaller than the male subgroup, and the numerically larger OS benefit observed in women warrants confirmation in sex-balanced external cohorts. Baseline-only MetS assessment may underestimate within-treatment metabolic-trajectory effects (Stable, Persistent, Incident, or Resolved MetS); a prespecified dynamic-MetS analysis is planned in the prospective validation cohort. Modest between-center quantitative heterogeneity was detected (center × MetS P-interaction , 0.018; I² , 55%), driven by one center with only three pCR events in the MetS subgroup. Internal-external validation by temporal split and leave-one-center-out indicated robust discrimination (AUC range 0.61–0.78), but prospective external validation in independent multicenter cohorts remains essential. Finally, H. pylori was assessed only as binary status; virulence-factor genotyping (CagA, VacA s1m1) and intra-tumoural microbiome profiling are planned in the prospective cohort. Three additional limitations should be acknowledged. First, although we adjusted for the number of preoperative PD-1 inhibitor administrations and chemotherapy cycles, treatment exposure in retrospective studies may capture multiple unmeasured clinical processes (planned regimen intensity, treatment tolerance, early discontinuation, physician preference), and residual confounding by treatment intensity cannot be fully excluded. Second, the five participating centers used harmonised but locally implemented pathology workflows; central pathology review of TRG, ypT/ypN, MSI, and PD-L1 CPS would strengthen reproducibility. Third, the MetS-inclusive nomogram was internally validated only; external validation in independent multicenter cohorts and prospective testing in randomised neoadjuvant immunotherapy trials are essential next steps before clinical deployment.

In conclusion, this study demonstrates that MetS is an independent favorable predictor of pathological response and survival in LAGC patients receiving neoadjuvant immunochemotherapy, without significantly amplifying irAE risk. A positive dose-response between MetS component burden and pCR probability, combined with RCS-identified metabolic sweet spots, supports an immunometabolic priming model for ICI enhancement in the perioperative context. The MetS-inclusive nomogram offers improved individualized pCR prediction. These findings motivate prospective validation in randomized trial substudies and motivate prospective external validation in independent multicenter cohorts and randomized trial substudies.

## Data Availability

The data analyzed in this study is subject to the following licenses/restrictions: The participant data with identifiers used to support the findings of this study were supplied by Qun Zhao under license and thus cannot be made freely available. The requests for access to these data should be made to Qun Zhao, zhaoqun@hebmu.edu.cn. Requests to access these datasets should be directed to Qun Zhao, zhaoqun@hebmu.edu.cn.

## References

[1] Sung H Ferlay J Siegel RL Laversanne M Soerjomataram I Jemal A . Global cancer statistics 2020: GLOBOCAN estimates of incidence and mortality worldwide for 36 cancers in 185 countries. CA Cancer J Clin. (2021) 71:209–49. doi: 10.3322/caac.21660 33538338

[2] Wang FH Zhang XT Tang L Wu Q Cai MY Li YF . The Chinese Society of Clinical Oncology (CSCO): Clinical guidelines for the diagnosis and treatment of gastric cancer, 2023. Cancer Commun (Lond). (2024) 44:127–72. doi: 10.1002/cac2.12516 38160327 PMC10794017

[3] Al-Batran SE Homann N Pauligk C Goetze TO Meiler J Kasper S . Perioperative chemotherapy with fluorouracil plus leucovorin, oxaliplatin, and docetaxel versus fluorouracil or capecitabine plus cisplatin and epirubicin for locally advanced, resectable gastric or gastro-oesophageal junction adenocarcinoma (FLOT4): a randomised, phase 2/3 trial. Lancet. (2019) 393:1948–57. doi: 10.1016/S0140-6736(18)32557-1 30982686

[4] Lorenzen S Götze TO Thuss-Patience P Biebl M Homann N Schenk M . Perioperative atezolizumab plus FLOT for resectable esophagogastric cancer: Interim results from the DANTE/IKF-s633 trial. J Clin Oncol. (2024) 42:410–20. doi: 10.1200/jco.23.00975 37963317

[5] Janjigian YY Shitara K Moehler M Garrido M Salman P Shen L . First-line nivolumab plus chemotherapy versus chemotherapy alone for advanced gastric, gastro-oesophageal junction, and oesophageal adenocarcinoma (CheckMate 649). Lancet. (2021) 398:27–40. doi: 10.1016/s0140-6736(21)00797-2 34102137 PMC8436782

[6] Shitara K Van Cutsem E Bang YJ Fuchs C Wyrwicz L Lee KW . Efficacy and safety of pembrolizumab or pembrolizumab plus chemotherapy vs chemotherapy alone for patients with first-line, advanced gastric cancer: The KEYNOTE-062 phase 3 randomized clinical trial. JAMA Oncol. (2020) 6:1571–80. doi: 10.1001/jamaoncol.2020.3370 32880601 PMC7489405

[7] Grundy SM Cleeman JI Daniels SR Donato KA Eckel RH Franklin BA . Diagnosis and management of the metabolic syndrome: An American Heart Association/National Heart, Lung, and Blood Institute scientific statement. Circulation. (2005) 112:2735–52. doi: 10.1161/CIRCULATIONAHA.105.169404 16157765

[8] Petrie JR Guzik TJ Touyz RM . Diabetes, hypertension, and cardiovascular disease: Clinical insights and vascular mechanisms. Can J Cardiol. (2018) 34:575–84. doi: 10.1016/j.cjca.2017.12.005 29459239 PMC5953551

[9] McQuade JL Daniel CR Hess KR Mak C Wang DY Rai RR . Association of body-mass index and outcomes in patients with metastatic melanoma treated with targeted therapy, immunotherapy, or chemotherapy: a retrospective, multicohort analysis. Lancet Oncol. (2018) 19:310–22. doi: 10.1016/s1470-2045(18)30078-0 29449192 PMC5840029

[10] Wang Z Aguilar EG Luna JI Dunai C Khuat LT Le CT . Paradoxical effects of obesity on T cell function during tumor progression and PD-1 checkpoint blockade. Nat Med. (2019) 25:141–51. doi: 10.1038/s41591-018-0221-5 30420753 PMC6324991

[11] Ringel AE Drijvers JM Baker GJ Catozzi A García-Cañaveras JC Gassaway BM . Obesity shapes metabolism in the tumor microenvironment to suppress anti-tumor immunity. Cell. (2020) 183:1848–1866.e26. doi: 10.1016/j.cell.2020.11.009 33301708 PMC8064125

[12] Cortellini A Bersanelli M Buti S Cannita K Santini D Perrone F . A multicenter study of body mass index in cancer patients treated with anti-PD-1/PD-L1 immune checkpoint inhibitors: when overweight becomes favorable. J Immunother Cancer. (2019) 7:57. doi: 10.1186/s40425-019-0527-y 30813970 PMC6391761

[13] Bou Zerdan M Ashok Kumar P Barrios DM Glidden A Nasr D Niforatos S . Metabolic syndrome is independently associated with improved overall survival to first-line therapy with immune checkpoint inhibitors in non-small cell lung cancer. Front Oncol. (2023) 13:1134824. doi: 10.3389/fonc.2023.1134824 37251929 PMC10213668

[14] Becker K Mueller JD Schulmacher C Ott K Fink U Busch R . Histomorphology and grading of regression in gastric carcinoma treated with neoadjuvant chemotherapy. Cancer. (2003) 98:1521–30. doi: 10.1002/cncr.11660 14508841

[15] Ma L Zhu W Wang Y Zhao L Wei X Xiong X . Association of the triglyceride-glucose index with immunotherapy efficacy and long-term prognosis in advanced non-small cell lung cancer. Front Oncol. (2025) 15:1616302. doi: 10.3389/fonc.2025.1616302 41079101 PMC12510814

[16] Alberti KG Eckel RH Grundy SM Zimmet PZ Cleeman JI Donato KA . Harmonizing the metabolic syndrome: a joint interim statement of the International Diabetes Federation Task Force on Epidemiology and Prevention; National Heart, Lung, and Blood Institute; American Heart Association; World Heart Federation; International Atherosclerosis Society; and International Association for the Study of Obesity. Circulation. (2009) 120:1640–5. doi: 10.1161/CIRCULATIONAHA.109.192644 19805654

[17] Lu J Wang L Li M Xu Y Jiang Y Wang W . Metabolic syndrome among adults in China: The 2010 China Noncommunicable Disease Surveillance. J Clin Endocrinol Metab. (2017) 102:507–15. doi: 10.1210/jc.2016-2477 27898293

[18] Eisenhauer EA Therasse P Bogaerts J Schwartz LH Sargent D Ford R . New response evaluation criteria in solid tumours: revised RECIST guideline (version 1.1). Eur J Cancer. (2009) 45:228–47. doi: 10.1016/j.ejca.2008.10.026 19097774

[19] Ding P Guo H Sun C Yang P Kim NH Tian Y . Combined systemic immune-inflammatory index (SII) and prognostic nutritional index (PNI) predicts chemotherapy response and prognosis in locally advanced gastric cancer patients receiving neoadjuvant chemotherapy with PD-1 antibody sintilimab and XELOX: a prospective study. BMC Gastroenterol. (2022) 22:121. doi: 10.1186/s12876-022-02199-9 35287591 PMC8919583

[20] Belsley DA Kuh E Welsch RE . Regression Diagnostics: Identifying Influential Data and Sources of Collinearity. New York: Wiley (1980).

[21] Rosenbaum PR Rubin DB . Constructing a control group using multivariate matched sampling methods that incorporate the propensity score. Am Stat. (1985) 39:33–8. doi: 10.1080/00031305.1985.10479383 41292463

[22] Collins GS Moons KGM Dhiman P Riley RD Beam AL Van Calster B . TRIPOD+AI statement: updated guidance for reporting clinical prediction models that use regression or machine learning methods. BMJ. (2024) 385:e078378. doi: 10.1136/bmj-2023-078378 38626948 PMC11019967

[23] Tchernof A Després JP . Pathophysiology of human visceral obesity: an update. Physiol Rev. (2013) 93:359–404. doi: 10.1152/physrev.00033.2011 23303913

[24] Conforti F Pala L Bagnardi V De Pas T Martinetti M Viale G . Cancer immunotherapy efficacy and patients' sex: a systematic review and meta-analysis. Lancet Oncol. (2018) 19:737–46. doi: 10.1016/s1470-2045(18)30261-4 29778737

[25] Pradhan AD . Sex differences in the metabolic syndrome: implications for cardiovascular health in women. Clin Chem. (2014) 60:44–52. doi: 10.1373/clinchem.2013.202549 24255079

[26] Chen S Ding P Yang Y Ma S Guo H Han X . Multimodal digital biopsy for preoperative prediction of occult peritoneal metastasis in gastric cancer. NPJ Digit Med. (2026) 9:107. doi: 10.1038/s41746-025-02268-9 41588105 PMC12865198

[27] Ding P Yang J Guo H Wu J Wu H Li T . Multimodal artificial intelligence-based virtual biopsy for diagnosing abdominal lavage cytology-positive gastric cancer. Adv Sci (Weinh). (2025) 12:e2411490. doi: 10.1002/advs.202411490 39985379 PMC12005817

[28] Ding P Chen S Guo H Yang J Fu Q Wang B . Radiomics-based ensemble model predicts postoperative recurrence of gastric cancer. BMC Med. (2025) 23:656. doi: 10.1186/s12916-025-04393-4 41291636 PMC12648815

[29] Ding P Yang J Chen S Guo H Wu J Wu H . Interpretable multimodal fusion model enhances postoperative recurrence prediction in gastric cancer. Adv Sci (Weinh). (2025) 12:e08190. doi: 10.1002/advs.202508190 40944934 PMC12631903

[30] Ding P Chen S Guo H Yang S Wang X Han X . A deep learning-based digital biopsy for predicting early recurrence in gastric cancer. Nat Commun. (2026) 17:5220. doi: 10.1038/s41467-026-71347-6 41986314 PMC13261139

[31] Albiges L Hakimi AA Xie W McKay RR Simantov R Lin X . Body mass index and metastatic renal cell carcinoma: Clinical and biological correlations. J Clin Oncol. (2016) 34:3655–63. doi: 10.1200/jco.2016.66.7311 27601543 PMC5065111

[32] Myers MG Leibel RL Seeley RJ Schwartz MW . Obesity and leptin resistance: distinguishing cause from effect. Trends Endocrinol Metab. (2010) 21:643–51. doi: 10.1016/j.tem.2010.08.002 20846876 PMC2967652

