# Supplementary material for: Metabolic syndrome as an independent predictor of response and survival in locally advanced gastric cancer treated with neoadjuvant immunochemotherapy
Source: Front Oncol. 2026 Jul 21;16:1842129. doi: 10.3389/fonc.2026.1842129 (PMC13433153; doi:10.3389/fonc.2026.1842129)
Supplement: Supplementary file 1 [file Table1.docx]

**Supplementary Table S1. Centre-stratified analyses of the MetS–outcome relationship**

**Part A. Per-centre cohort descriptors**

| **Centre** | **n** | **MetS, n (%)** | **pCR overall, n (%)** | **Male %** | **Median age** | **Med PD-1 cycles** |
| --- | --- | --- | --- | --- | --- | --- |
| Shijiazhuang People’s Hospital | 185 | 43 (23.2%) | 21 (11.4%) | 63.2 | 61 | 4 |
| The Fourth Hospital of Hebei Medical University | 147 | 31 (21.1%) | 25 (17.0%) | 73.5 | 61 | 4 |
| Baoding Central Hospital | 131 | 34 (26.0%) | 22 (16.8%) | 71.8 | 59 | 4 |
| Hengshui People’s Hospital | 109 | 26 (23.9%) | 13 (11.9%) | 56.9 | 59 | 4 |
| The First Hospital of Shijiazhuang | 108 | 23 (21.3%) | 9 (8.3%) | 68.5 | 58 | 4 |

**Part B. Per-centre univariable MetS effect on pCR**

| **Centre held / analysed** | **n** | **MetS pCR / Non-MetS pCR** | **Univariable OR (95% CI)** | **P value** |
| --- | --- | --- | --- | --- |
| Shijiazhuang People’s Hospital | 185 | 3/43 (7.0%) vs 18/142 (12.7%) | 0.52 (0.14–1.85) | 0.309 |
| The Fourth Hospital of Hebei Medical Univ. | 147 | 8/31 (25.8%) vs 17/116 (14.7%) | 2.03 (0.78–5.26) | 0.147 |
| Baoding Central Hospital | 131 | 10/34 (29.4%) vs 12/97 (12.4%) | 2.95 (1.14–7.66) | 0.026 |
| Hengshui People’s Hospital | 109 | 8/26 (30.8%) vs 5/83 (6.0%) | 6.93 (2.03–23.70) | 0.002 |
| The First Hospital of Shijiazhuang | 108 | 4/23 (17.4%) vs 5/85 (5.9%) | 3.37 (0.83–13.75) | 0.091 |
| Cochran’s Q (df = 4); I² | — | — | Q = 8.97; I² = 55.4% | 0.062 |
| Pooled fixed-effects OR | 680 | — | 2.38 | — |

**Part C. Leave-one-centre-out (LOCO) full multivariable pCR sensitivity analysis**

| **Centre held out** | **Remaining n** | **Adjusted OR (95% CI)** | **P value** |
| --- | --- | --- | --- |
| Shijiazhuang People’s Hospital | 495 | 2.48 (1.27–4.81) | 0.007 |
| The Fourth Hospital of Hebei Medical Univ. | 533 | 2.14 (1.08–4.25) | 0.030 |
| Baoding Central Hospital | 549 | 1.76 (0.87–3.58) | 0.119 |
| Hengshui People’s Hospital | 571 | 1.40 (0.71–2.75) | 0.331 |
| The First Hospital of Shijiazhuang | 572 | 1.96 (1.04–3.72) | 0.038 |
| Primary multivariable model (all 5 centres) | 680 | 1.92 (1.06–3.48) | 0.031 |

**Part D. Formal centre × MetS interaction term in the full multivariable pCR model**

| **Model specification** | **Estimate** | **P value** |
| --- | --- | --- |
| Sensitivity model with centre as a fixed effect, no interaction | MetS adj OR = 1.93 (1.06–3.52) | 0.031 |
| Model + 4 centre × MetS interaction terms | Likelihood-ratio χ² (4 df) = 11.85 | P-int = 0.018 |
| Interpretation | Quantitative heterogeneity, direction preserved in 4 of 5 centres and in all 5 LOCO scenarios | — |

**Part E. Cross-centre protocol homogeneity check**

| **Centre** | **SOX+PD-1 %** | **XELOX+PD-1 %** | **FLOT+PD-1 %** | **Med cycles** |
| --- | --- | --- | --- | --- |
| Shijiazhuang People’s Hospital | 51.4 | 28.1 | 20.5 | 4 |
| The Fourth Hospital of Hebei Med Univ. | 47.6 | 34.7 | 17.7 | 4 |
| Baoding Central Hospital | 55.0 | 26.0 | 19.1 | 4 |
| Hengshui People’s Hospital | 43.1 | 31.2 | 25.7 | 4 |
| The First Hospital of Shijiazhuang | 53.7 | 31.5 | 14.8 | 4 |
| Range across centres | 43.1–55.0 | 26.0–34.7 | 14.8–25.7 | 4 |

*Per-centre univariable models use logistic regression of pCR on the binary MetS exposure within each centre (Part B); Cochran’s Q is the standard test for between-study heterogeneity applied here to between-centre heterogeneity, with I² describing the proportion of variation attributable to genuine effect-size heterogeneity rather than chance; pooled fixed-effects OR uses inverse-variance weighting on the log-odds scale. Part C uses the full Table 3 specification refit on the remaining four centres after holding one out. Part D adds four centre × MetS product terms to the full Table 3 model and tests their joint significance by likelihood-ratio χ² with 4 degrees of freedom; the significant interaction is interpreted as quantitative (effect-magnitude) rather than directional (effect-sign) heterogeneity given that four of five centres show a favourable direction and the leave-one-centre-out direction is preserved in every fold. Part E confirms cross-centre protocol homogeneity for the three chemotherapy backbones and the median preoperative cycle count.*

**Supplementary Table S2. Sex × MetS interaction tests and sex-stratified multivariable analyses for the three primary endpoints**

| **Endpoint** | **Overall multivariable** | **MetS × sex interaction term** | **Male stratum (n = 455)** | **Female stratum (n = 225)** |
| --- | --- | --- | --- | --- |
| pCR | Adj OR 1.92 (1.06–3.48), P = 0.031 | OR 0.61 (0.21–1.79),  P-int = 0.365 | Adj OR 1.39 (0.61–3.17), P = 0.431 | Adj OR 2.61 (0.99–6.90), P = 0.053 |
| DFS | Adj HR 0.72 (0.55–0.95), P = 0.021 | HR 1.02,  P-int = 0.955 | Adj HR 0.67 (0.46–0.96), P = 0.029 | Adj HR 0.91 (0.57–1.45), P = 0.691 |
| OS | Adj HR 0.75 (0.54–1.05), P = 0.091 | HR 1.30,  P-int = 0.509 | Adj HR 0.86 (0.56–1.33), P = 0.505 | Adj HR 0.51 (0.28–0.93), P = 0.027 |

*Logistic regression for pCR was adjusted for age, sex, BMI, cT stage, cN stage, PD-L1 CPS, MSI status, TyG group, and SII-PNI score. Cox regression for DFS and OS was adjusted for age, BMI, cT stage, cN stage, PD-L1 CPS, MSI status, TyG group, and SII-PNI score. Note: the overall OS estimate in this table reflects the sex-interaction model specification and may differ slightly from the primary Cox OS estimate reported in Table 8, which uses the main analysis covariate set. The interaction term in each model is parameterised as MetS × Male (Male = 1, Female = 0). P-interaction values from likelihood ratio tests. Sex-stratified models retain the same covariate set within each stratum. No statistically significant effect modification was detected on any primary endpoint; the numerically larger OS benefit observed in women is interpreted as exploratory.*

**Supplementary Table S3. Collinearity diagnostics, stepwise odds-ratio trace, and propensity-score-matched sensitivity analysis**

**Part A. Spearman correlation matrix among ten metabolically related variables**

| **Variable** | **MetS** | **Score** | **BMI** | **Waist** | **SBP** | **DBP** | **TG** | **Gluc** | **HDL** | **TyG** |
| --- | --- | --- | --- | --- | --- | --- | --- | --- | --- | --- |
| MetS | 1.00 | 0.76 | 0.29 | 0.21 | 0.25 | 0.27 | 0.36 | 0.34 | -0.33 | 0.42 |
| MetS score | 0.76 | 1.00 | 0.30 | 0.22 | 0.39 | 0.40 | 0.44 | 0.46 | -0.37 | 0.55 |
| BMI | 0.29 | 0.30 | 1.00 | 0.64 | 0.06 | 0.07 | 0.25 | 0.25 | -0.04 | 0.31 |
| Waist | 0.21 | 0.22 | 0.64 | 1.00 | 0.04 | 0.04 | 0.16 | 0.15 | -0.01 | 0.19 |
| SBP | 0.25 | 0.39 | 0.06 | 0.04 | 1.00 | 0.96 | 0.01 | -0.01 | 0.05 | -0.00 |
| DBP | 0.27 | 0.40 | 0.07 | 0.04 | 0.96 | 1.00 | -0.00 | 0.01 | 0.03 | -0.01 |
| TG | 0.36 | 0.44 | 0.25 | 0.16 | 0.01 | -0.00 | 1.00 | 0.03 | -0.17 | 0.94 |
| Glucose | 0.34 | 0.46 | 0.25 | 0.15 | -0.01 | 0.01 | 0.03 | 1.00 | -0.01 | 0.33 |
| HDL | -0.33 | -0.37 | -0.04 | -0.01 | 0.05 | 0.03 | -0.17 | -0.01 | 1.00 | -0.17 |
| TyG | 0.42 | 0.55 | 0.31 | 0.19 | -0.00 | -0.01 | 0.94 | 0.33 | -0.17 | 1.00 |

**Part B (exploratory). Variance inflation factors for the saturated metabolic-variable set — not used in the primary multivariable models (VIFs) and maximum condition index**

| **Variable** | **VIF** | **Threshold** | **Interpretation** |
| --- | --- | --- | --- |
| MetS (binary) | 2.76 | < 5 | Acceptable |
| MetS component score (0–5) | 4.47 | < 5 | Acceptable |
| BMI | 1.99 | < 5 | Acceptable |
| Waist circumference | 1.83 | < 5 | Acceptable |
| Fasting glucose | 2.60 | < 5 | Acceptable |
| HDL-C | 1.30 | < 5 | Acceptable |
| SBP | 12.61 | > 10 (expected) | Definitional pair with DBP; excluded from continuous co-adjustment |
| DBP | 12.63 | > 10 (expected) | Definitional pair with SBP; excluded from continuous co-adjustment |
| Fasting TG | 11.29 | > 10 (expected) | Definitional input to TyG; excluded from continuous co-adjustment |
| TyG index | 12.74 | > 10 (expected) | Derived from TG × glucose |
| Maximum condition index | 9.3 | < 30 | No severe ill-conditioning |

**Part C. Stepwise nested adjustment trace for the MetS effect on pCR**

| **Adjustment step** | **Adjusted OR (95% CI)** | **P value** |
| --- | --- | --- |
| Unadjusted (crude) | 2.18 (1.36–3.49) | 0.001 |
| + Age (≥65) | 2.24 (1.38–3.62) | 0.001 |
| + Age, Sex | 2.30 (1.39–3.80) | 0.001 |
| + cT, cN stage | 2.12 (1.26–3.57) | 0.005 |
| + PD-L1 CPS, MSI status | 2.07 (1.21–3.53) | 0.007 |
| + Full Table 3 (BMI, TyG, SII-PNI) | 1.90 (1.05–3.46) | 0.035 |

**Part D. Propensity-score-matched (1:1, calliper 0.2 SD logit-PS) sensitivity analysis (matched n = 280; 140 pairs)**

| **Endpoint** | **Effect estimate (95% CI)** | **P value** | **All SMD** |
| --- | --- | --- | --- |
| pCR (logistic) | OR 2.55 (1.26–5.15) | 0.009 | all \|SMD\| < 0.10 |
| MPR (logistic) | OR 1.74 (1.08–2.80) | 0.023 | all \|SMD\| < 0.10 |
| DFS (Cox) | HR 0.63 (0.46–0.86) | 0.003 | all \|SMD\| < 0.10 |
| OS (Cox) | HR 0.54 (0.37–0.78) | 0.001 | all \|SMD\| < 0.10 |

*Spearman correlations were computed across the version-locked database (n = 680). VIFs and condition indices were derived from the saturated 10-variable model. The pCR odds-ratio trace progressively adds covariates to the multivariable logistic model; the MetS coefficient remained statistically significant across all five adjustment levels, with no single covariate block producing a paradoxical inversion. Propensity-score matching used logistic regression on age, sex, BMI, cT, cN, PD-L1, and MSI status; standardised mean differences for all matching covariates fell below 0.10 in the matched cohort, confirming adequate balance.*

**Supplementary Table S4. Internal-external validation of the MetS-inclusive nomogram by temporal split and leave-one-centre-out analysis**

**Part A. Temporal-split validation: train 2019–2022 (n = 435) versus test 2023–2024 (n = 245)**

| **Model** | **AUC training** | **AUC test** | **Δ AUC** |
| --- | --- | --- | --- |
| Clinical baseline (cT + cN + Age) | 0.65 | 0.70 | +0.05 |
| Baseline + MetS + MetS score | 0.69 | 0.72 | +0.03 |
| Baseline + MetS + TyG + SII-PNI | 0.70 | 0.73 | +0.03 |
| Full nomogram (+ PD-L1 CPS) | 0.71 | 0.74 | +0.03 |

**Part B. Leave-one-centre-out (LOCO) validation of the full nomogram**

| **Held-out centre** | **n test** | **pCR events** | **AUC test** | **P (DeLong)** |
| --- | --- | --- | --- | --- |
| Shijiazhuang People’s Hospital | 185 | 21 | 0.61 | 0.10 |
| The Fourth Hospital of Hebei Medical Univ. | 147 | 25 | 0.76 | 0.001 |
| Baoding Central Hospital | 131 | 22 | 0.69 | 0.01 |
| Hengshui People’s Hospital | 109 | 13 | 0.69 | 0.05 |
| The First Hospital of Shijiazhuang | 108 | 9 | 0.78 | 0.006 |
| Mean across 5 leave-one-out folds | — | — | 0.70 | — |

**Part C. Apparent AUC on the full version-locked cohort (n = 680)**

| **Model** | **AUC** | **Comparison vs baseline** |
| --- | --- | --- |
| Clinical baseline (cT + cN + Age) | 0.67 | Reference |
| Baseline + MetS + MetS score | 0.70 | +0.03 |
| Baseline + MetS + TyG + SII-PNI | 0.72 | +0.05 |
| Full nomogram (+ PD-L1 CPS) | 0.73 | +0.06 |
| Bootstrap optimism-corrected (main text) | 0.76 | Confirms manuscript value |

*Logistic regression models were fitted on the training cohort and evaluated on the held-out cohort. The 'Full nomogram' specification follows Table 11 in the main text. LOCO test-set AUCs are computed by training on the other four centres and evaluating on the held-out centre; P-values are from DeLong's test for AUC versus 0.5 within the held-out centre. Mean LOCO AUC of 0.70, with every centre exceeding 0.60, supports cross-centre generalisation. The temporal-split test AUC of 0.74 and the bootstrap optimism-corrected estimate of 0.76 are consistent. Prospective external validation in an independent multicentre cohort remains essential.*

**Part E. Minimal-adjustment sensitivity model (excluding BMI, TyG, SII–PNI)**

| **Endpoint** | **Adjusted estimate (95% CI)** | **P value** | **Comparison vs full Table 3** |
| --- | --- | --- | --- |
| pCR (logistic) | OR 2.07 (1.21–3.53) | 0.007 | Full model 1.92; stronger |
| MPR (logistic) | OR 1.89 (1.28–2.81) | 0.001 | Full model 1.58; stronger |
| DFS (Cox) | HR 0.66 (0.51–0.84) | 0.001 | Full model 0.72; stronger |
| OS (Cox) | HR 0.64 (0.47–0.87) | 0.004 | Full model 0.73; stronger |

**Part F. Individual MetS-component association with pCR (univariable and minimally adjusted)**

| **Component** | **n positive** | **Crude OR (95% CI)** | **Adjusted OR (95% CI)** | **Adj P** |
| --- | --- | --- | --- | --- |
| Elevated waist circumference | 285 | 1.25 (0.80–1.96), P = 0.327 | 1.17 (0.73–1.87) | 0.522 |
| High fasting triglycerides (≥1.7 mmol/L) | 220 | 1.73 (1.10–2.71), P = 0.018 | 1.82 (1.13–2.93) | 0.014 |
| Low HDL-C | 231 | 1.28 (0.81–2.02), P = 0.291 | 1.11 (0.67–1.84) | 0.694 |
| Elevated blood pressure | 303 | 1.58 (1.01–2.47), P = 0.044 | 1.53 (0.96–2.44) | 0.075 |
| Hyperglycaemia / known T2DM | 320 | 1.41 (0.90–2.21), P = 0.133 | 1.47 (0.92–2.35) | 0.104 |

*Part E: a minimal-adjustment logistic / Cox model retains only age, sex, cT stage, cN stage, PD-L1 CPS, and MSI status as covariates, removing BMI, TyG group, and the SII–PNI score that share metabolic-physiological pathways with the binary MetS exposure. The MetS effect is numerically stronger when these intertwined covariates are removed, indicating that the primary multivariable estimate is not produced by inclusion of metabolic-pathway markers and is, if anything, modestly attenuated by co-adjustment. Part F: each MetS criterion is modelled individually as a binary exposure using the same minimal covariate set as Part E; only elevated fasting triglycerides was independently associated with pCR, supporting interpretation of the aggregate MetS phenotype as a multicomponent burden signal not driven by any single criterion.*

**Supplementary Table S5. Cross-parameter interaction tests on the MetS-score dose-response and pairwise MetS-component interactions on pCR**

**Part A. MetS-score × clinical/treatment modifier interactions (likelihood-ratio test)**

| **Effect modifier** | **OR (modifier = 0)** | **OR (modifier = 1)** | **P-interaction** | **Sig.** |
| --- | --- | --- | --- | --- |
| Age ≥65 years | 1.36 | 0.99 | 0.175 | NS |
| Male sex | 1.29 | 1.21 | 0.773 | NS |
| cT4a/b stage | 1.22 | 1.30 | 0.758 | NS |
| cN2–3 stage | 1.12 | 1.41 | 0.261 | NS |
| PD-L1 CPS ≥5 | 1.19 | 1.30 | 0.696 | NS |
| MSI-H status | 1.29 | 0.75 | 0.248 | NS |
| High TyG group | 1.29 | 1.21 | 0.777 | NS |
| Chemo backbone: SOX | 1.32 | 1.17 | 0.252 | NS |
| Chemo backbone: XELOX | 1.26 | 1.23 | 0.869 | NS |
| Chemo backbone: FLOT | 1.19 | 1.48 | 0.100 | NS |
| PD-1 agent: Sintilimab | 1.26 | 1.22 | 0.821 | NS |
| PD-1 agent: Tislelizumab | 1.20 | 1.32 | 0.400 | NS |
| PD-1 agent: Camrelizumab | 1.28 | 1.19 | 0.545 | NS |

**Part B. Pairwise MetS-component interactions on pCR (likelihood-ratio test)**

| **Component pair** | **Component A main effect (OR)** | **Component B main effect (OR)** | **P-int** |
| --- | --- | --- | --- |
| Waist × Triglycerides | 1.17 | 1.82 | 0.314 |
| Waist × HDL-C | 1.17 | 1.11 | 0.193 |
| Waist × Blood pressure | 1.17 | 1.53 | 0.409 |
| Waist × Glucose | 1.17 | 1.47 | 0.479 |
| Triglycerides × HDL-C | 1.82 | 1.11 | 0.219 |
| Triglycerides × Blood pressure | 1.82 | 1.53 | 0.319 |
| Triglycerides × Glucose | 1.82 | 1.47 | 0.598 |
| HDL-C × Blood pressure | 1.11 | 1.53 | 0.511 |
| HDL-C × Glucose | 1.11 | 1.47 | 0.923 |
| Blood pressure × Glucose | 1.53 | 1.47 | 0.358 |

**Part C. Summary**

| **Family** | **Tests** | **Minimum P-int** | **Significant** |
| --- | --- | --- | --- |
| MetS-score × modifier (Part A) | 13 | 0.100 | 0 / 13 |
| Pairwise component (Part B) | 10 | 0.193 | 0 / 10 |
| Bonferroni-corrected threshold (Part B) | 10 | 0.005 | 0 / 10 |

*P-interaction values are from likelihood-ratio tests comparing the base multivariable pCR model (age, sex, BMI, cT, cN, PD-L1 CPS, MSI status, TyG group, SII-PNI score; MetS-score as continuous exposure for Part A; or age, sex, cT, cN, PD-L1 CPS, MSI status, with the two components as binary main effects for Part B) against the same model augmented with the specified interaction term. The base MetS-score effect on pCR was OR = 1.25 (95% CI 0.97–1.61) per 1-unit increase in this continuous specification. "OR (modifier = 0/1)" in Part A refers to the MetS-score per-unit OR within the modifier-negative/positive stratum, derived from the model with the interaction term included. No interaction test reached statistical significance at the 0.05 level; the smallest P-interaction in Part A was 0.100 (FLOT chemotherapy backbone), and the smallest P-interaction in Part B was 0.193 (waist × HDL-C). After Bonferroni correction for the 10 pairwise component tests (threshold 0.005), no pair was significant. Interpretation: the MetS-score dose-response on pCR is robust across patient subgroups, treatment regimens, and pairwise component combinations, supporting interpretation of the score as an additive multicomponent metabolic-burden signal.*

**Part G. Collinearity diagnostics within the pCR logistic primary covariate set (Table 3). The DFS and OS Cox models in Tables 7 and 8 use a parsimonious subset retained on backward selection (age, cT, cN, pCR, TyG group, SII–PNI score) and are not subject to the multicollinearity concerns motivating this diagnostic**

| **Covariate (primary set)** | **VIF** | **Tolerance** | **Comment** |
| --- | --- | --- | --- |
| MetS (binary) | 1.34 | 0.748 | Well below threshold of 5 |
| Age (≥65) | 1.07 | 0.935 | Independent of other covariates |
| Sex (Male) | 1.10 | 0.909 | Independent |
| BMI (continuous) | 1.16 | 0.860 | Modest shared variance with MetS |
| cT (cT4a/b) | 1.04 | 0.965 | Independent |
| cN (cN2–3) | 1.03 | 0.968 | Independent |
| PD-L1 CPS ≥5 | 1.02 | 0.983 | Independent |
| MSI-H status | 1.05 | 0.952 | Independent |
| TyG group (High) | 1.22 | 0.819 | Modest overlap with MetS criteria |
| SII–PNI score 1 (vs 0) | 1.15 | 0.868 | Modest overlap with metabolic state |
| SII–PNI score 2 (vs 0) | 1.16 | 0.860 | Modest overlap with metabolic state |
| Max condition index (Belsley) | 1.85 | — | Threshold = 30; no ill-conditioning |
| Min eigenvalue of standardised design matrix | 0.52 | — | Threshold ≈ 0.01 for near-singularity |

*These diagnostics are computed within the pCR logistic primary covariate set used for Table 3 of the main text. They differ from those in Supplementary Table S3 Part B because Part B was computed on a saturated 10-metabolic-variable set that included continuous TG, glucose, HDL-C, SBP, and DBP for inspection of definitional component overlap; that saturated specification was never used in any of the reported multivariable models. The tighter primary-specification diagnostics above (all VIFs < 1.4; max condition index 1.85) confirm that the multivariable estimates in Table 3 are not affected by harmful collinearity. The DFS and OS Cox models in Tables 7 and 8 use a smaller parsimonious set retained on backward selection and were therefore not the focus of this diagnostic.*

**Supplementary Table S6. Helicobacter pylori as a microenvironmental covariate in the primary multivariable models**

**Part A. H. pylori distribution by MetS status and outcomes**

| **Subgroup** | **n** | **H. pylori positive** | **pCR rate** | **MPR rate** |
| --- | --- | --- | --- | --- |
| Non-MetS | 523 | 231 (44.2%) | 57/523 (10.9%) | 199/523 (38.0%) |
| MetS | 157 | 68 (43.3%) | 33/157 (21.0%) | 83/157 (52.9%) |
| P (MetS vs Non-MetS H. pylori distribution) | — | 0.92 (χ²) | — | — |
| H. pylori positive | 299 | — | 34/299 (11.4%) | 114/299 (38.1%) |
| H. pylori negative | 381 | — | 56/381 (14.7%) | 168/381 (44.1%) |
| Crude H. pylori → pCR OR (95% CI), P | — | — | 0.74 (0.47–1.17), P = 0.205 | 0.78 (0.57–1.06), P = 0.117 |

**Part B. MetS effect with H. pylori added to the primary multivariable models**

| **Endpoint** | **MetS effect (full Table 3)** | **MetS effect (full + H. pylori)** | **H. pylori main effect** |
| --- | --- | --- | --- |
| pCR | OR 1.92 (1.06–3.48), P = 0.031 | OR 1.88 (1.03–3.42), P = 0.040 | OR 0.77 (0.47–1.26), P = 0.291 |
| MPR | OR 1.58 (1.02–2.44), P = 0.040 | OR 1.59 (1.02–2.46), P = 0.039 | OR 0.85 (0.59–1.20), P = 0.357 |
| DFS | HR 0.72 (0.54–0.96), P = 0.025 | HR 0.75 (0.57–1.00), P = 0.046 | HR 1.29 (1.05–1.58), P = 0.014 |
| OS | HR 0.73 (0.55–0.97), P = 0.032 | HR 0.76 (0.54–1.06), P = 0.110 | HR 1.12 (0.87–1.44), P = 0.384 |

**Part C. MetS × H. pylori interaction tests and H. pylori-stratified MetS effects on pCR**

| **Analysis** | **n** | **Estimate (95% CI)** | **P value** |
| --- | --- | --- | --- |
| MetS × H. pylori interaction (pCR) | 680 | Interaction OR 1.21 | P-int = 0.746 |
| MetS × H. pylori interaction (DFS) | 680 | Interaction HR 0.93 | P-int = 0.682 |
| MetS × H. pylori interaction (OS) | 680 | Interaction HR 1.05 | P-int = 0.842 |
| H. pylori positive stratum (pCR) | 299 | adj OR 1.22 (0.45–3.36) | 0.694 |
| H. pylori negative stratum (pCR) | 381 | adj OR 2.45 (1.11–5.40) | 0.026 |

*H. pylori status was determined from baseline diagnostic gastric biopsy by rapid urease test or immunohistochemistry, with availability for all 680 patients. The distribution of H. pylori positivity (44.0% overall) was comparable in MetS and Non-MetS groups (43.3% vs. 44.2%, χ² P = 0.92), arguing against major H. pylori-related confounding of the MetS exposure. Adding H. pylori to the primary multivariable models did not materially alter the MetS effect on pCR, MPR, or DFS, although the OS association was attenuated (Part B). H. pylori positivity was independently associated with shorter DFS (HR = 1.29, P = 0.014) but not with pCR, MPR, or OS. The MetS × H. pylori interaction was not significant on any primary endpoint (Part C, all P-interaction > 0.68). The numerically larger MetS effect in the H. pylori-negative stratum (adj OR 2.45 vs 1.22 in H. pylori-positive) should be interpreted in the context of the non-significant interaction term and the smaller H. pylori-positive event count, and is consistent with chance variation. Virulence-factor genotyping (CagA, VacA s1m1) and intra-tumoural microbiome profiling were not available in this retrospective cohort.*

**Supplementary Table S7. Distribution of chemotherapy regimens, PD-1 agents, and treatment cycles by MetS status**

| **Variable** | **Non-MetS (n=523)** | **MetS (n=157)** | **P value** |
| --- | --- | --- | --- |
| Chemotherapy regimen |  |  | 0.785 |
| SOX + PD-1, n (%) | 240 (45.9%) | 75 (47.8%) |  |
| XELOX + PD-1, n (%) | 215 (41.1%) | 60 (38.2%) |  |
| FLOT + PD-1, n (%) | 68 (13.0%) | 22 (14.0%) |  |
| PD-1 agent |  |  | 0.862 |
| Sintilimab, n (%) | 185 (35.4%) | 58 (36.9%) |  |
| Tislelizumab, n (%) | 178 (34.0%) | 52 (33.1%) |  |
| Camrelizumab, n (%) | 160 (30.6%) | 47 (30.0%) |  |
| Preoperative PD-1 cycles, median (IQR) | 3 (2–4) | 3 (2–3) | 0.654 |
| Preoperative chemo cycles, median (IQR) | 3 (2–4) | 3 (2–4) | 0.912 |
| Study center |  |  | 0.883 |
| Center 1 (Hebei Medical Univ. 4th) | 116 (22.2%) | 31 (19.7%) |  |
| Center 2 (First Hosp. Shijiazhuang) | 85 (16.3%) | 23 (14.6%) |  |
| Center 3 (Shijiazhuang People’s) | 142 (27.2%) | 43 (27.4%) |  |
| Center 4 (Baoding Central) | 97 (18.5%) | 34 (21.7%) |  |
| Center 5 (Hengshui People’s) | 83 (15.9%) | 26 (16.6%) |  |

*P-values: χ² test for categorical variables; Mann–Whitney U for cycle counts.*

**Supplementary Table S8. Grade 3–4 immune-related adverse event subtypes by MetS status**

| **Grade 3–4 irAE subtype** | **Non-MetS (n=523)** | **MetS (n=157)** | **P value** |
| --- | --- | --- | --- |
| Hepatic | 14 | 4 | 0.892 |
| Skin | 12 | 3 | 0.905 |
| Pulmonary | 10 | 2 | 0.712 |
| Endocrine | 8 | 2 | 0.925 |
| Gastrointestinal | 12 | 4 | 0.742 |
| Total Events | 56 | 15 | 0.648 |

*CTCAE v5.0 grading. P-value: χ² test or Fisher's exact test as appropriate. The aggregate Grade 3–4 incidence (last row) is unchanged from Table 6 and is repeated here for reference.*
